# Rapid detection methods for foodborne pathogens based on nucleic acid amplification: Recent advances, remaining challenges, and possible opportunities

**DOI:** 10.1016/j.fochms.2023.100183

**Published:** 2023-09-18

**Authors:** Nodali Ndraha, Hung-Yun Lin, Chen-Yow Wang, Hsin-I Hsiao, Han-Jia Lin

**Affiliations:** a Department of Bioscience and Biotechnology, National Taiwan Ocean University, Keelung 202301, Taiwan; b Center of Excellence for the Oceans, National Taiwan Ocean University, Keelung 202301, Taiwan; c Department of Food Science, National Taiwan Ocean University, Keelung, 202301 Taiwan

**Keywords:** Polymerase chain reaction, Foodborne pathogen, Molecular detection method, Isothermal amplification, Thermo-cycle amplification

## Abstract

This article presents a review of recent advancements in the utilization of NAA-based techniques for detecting foodborne pathogens in food products, focusing on studies conducted within the past five years. This review revealed that recent research efforts have primarily aimed at enhancing sensitivity and specificity by improving sample pre-treatment/preparation, DNA isolation, and readout methods. Isothermal-based amplification methods, such as LAMP, RPA, RAA, and RCA, have emerged as promising approaches, providing rapid results within one h and often demonstrating comparable or superior sensitivity to conventional or qPCR methods. However, the attention paid to specific pathogens varies, with *Salmonella* spp., *Listeria* spp., *E. coli*, and *V. parahaemolyticus* receiving more focus than norovirus and other similar pathogens. NAA-based methods have the potential to significantly contribute to food safety and public health protection. However, further advancements are necessary to fully realize their benefits.

## Highlights

• Review of food safety analysis enabled by nucleic acid amplification (NAA)-based methods.
• Review of the applicability of NAA-based methods for on-site testing.
• Identification of prospects and technical challenges in NAA-based methods.
• Discussion on promoting widespread implementation of NAA-based methods.

## 1 Introduction

Foodborne illnesses caused by bacterial pathogens are a significant public health concern worldwide. The World Health Organization (WHO) estimates that these diseases result in approximately 600 million illnesses and 420,000 deaths annually, with the majority occurring in low- and middle-income countries with poor hygiene controls (WHO, 2022). Children under five years old are particularly vulnerable and account for almost one-third of foodborne illness-related deaths (WHO, 2022). Common pathogens include *Salmonella* spp., *Listeria* spp., *Escherichia coli*, and norovirus (WHO, 2022); this evidence highlights the importance of implementing effective food safety systems and continuously enhancing food safety on a global scale. To achieve this result, it is essential to develop rapid and accurate detection tools for detecting and identifying pathogens in food. These tools will facilitate preventive control and prompt corrective measures that will ultimately enhance the overall management of food safety (Panwar et al., 2022).

Traditionally, culture-dependent methods have been used for detecting and identifying pathogens in food products for decades. While these methods are generally simple and inexpensive, they can be time-consuming since they rely on the growth of the target pathogen, a process which can take up to 2–3 days for detection and up to 7 days for confirmation (Panwar et al., 2022, Zhu et al., 2020). Additionally, culture-based methods are limited in their ability to detect certain pathogens that are not grown in culture, are unable to distinguish between viable culturable cells and viable but non-culturable (VBNC) cells, and cannot provide sufficient information to differentiate among strains (Panwar et al., 2022, Zhu et al., 2020). Consequently, there is a need for more sensitive, rapid, and accurate detection methods.

The introduction of nucleic acid amplification (NAA)-based methods has revolutionized pathogen detection in the food industry (Panwar et al., 2022). These methods have gained popularity due to their ability to accurately identify pathogens. Recently, several researchers have studied and reviewed the principles, mechanisms, effectiveness, and applicability of NAA-based methods (Pang et al., 2022, Pumford et al., 2020, Xia et al., 2022). However, most of these reviews have primarily focused on isothermal amplification or specific food products, with limited discussion on how to enhance the widespread implementation of NAA-based methods, considering recent technological advancements. In this review, we aim to provide a comprehensive overview of the latest advancements in major NAA-based methods designed specifically for the detection of foodborne pathogens in food samples. Contributing factors that affect the performance of these methods are highlighted in this review. We additionally present a summary and comparison of the characteristics, strengths, and weaknesses of these methods; such analysis is crucial for obtaining a thorough understanding of the current state-of-the-art NAA-based methods, identifying implementation obstacles, and exploring potential solutions to overcome the challenges associated with these methods. Moreover, this review outlines recommendations aimed at improving the broad implementation of NAA-based methods in food testing, which will greatly contribute to enhancing food safety management efforts.

## 2 Recent advances in naa-based methods

In this review, we focused on enzyme-assisted NAA-based methods employed for the detection of foodborne pathogens in food samples; these include both thermo-cycle amplification and isothermal amplification techniques Table 1. In terms of thermo-cycle amplification methods, we focused on the recent advancements in conventional PCR, real-time PCR, and digital PCR. Regarding isothermal amplification, our focus extended to loop-mediated isothermal amplification (LAMP), recombinase polymerase amplification (RPA), recombinase aided amplification (RAA), rolling circle amplification (RCA), saltatory rolling circle amplification (SRCA), nucleic acid sequence-based amplification (NASBA), strand displacement amplification (SDA), exponential amplification reaction (EXPAR), single primer isothermal amplification (SPIA), helicase-dependent amplification (HDA), and cross priming amplification (CPA). We discussed and compared the characteristics of these methods based on their sensitivity, specificity, ease of use, and portability. Whenever available, we also considered additional aspects such as the total assay time and cost of operation. Examples of using these amplification methods in detecting pathogens in food samples are given below.

**Table 1:** Characteristics of NAA-based methods for detecting foodborne pathogens in food samples.

Amplification method	Primer	Enzyme	Temperature (°C)	Reaction time (min)	Reference
Conventional PCR	2	- DNA polymerase	50–94	50–120	Table S1
qPCR	2	- DNA polymerase, intercalating dyes or fluorescent probes	50–94	50–120	Table S2
Digital PCR	2	- Requires digestive or exonuclease enzymes - Requires fluorescent dyes and probes	50–94	50–120	(He et al., 2020, Lei et al., 2020)
LAMP	4–6	- *Bst* DNA polymerase	60–65	30–60	Table S3
RPA/RAA	2	- Recombinase protein, - Single stranded DNA binding protein TP32 (SSB) - DNA polymerase	37–42	20–40	Table S4–5
RCA	1–2	- Padlock probe - *Phi* DNA polymerase	37–65	60–120	Table S6
SRCA	2	- *Bst* DNA polymerase	60–70	20–80	Table S7
NASBA	2	- AVM RTase - T7 RNA polymerase - RNAse H	41	90–120	Table S8
SDA	2–4	- *exo*-Klenow fragment polymerase - REase HincII	25–50	20–120	Table S8
EXPAR	NR*	- *Bst* DNA polymerase, - Nicking endonuclease - One exponentially template	60	30–60	Table S8
SPIA	1	- Single and gene-specific DNA-RNA chimeric primer - DNA polymerase with strong strand-displacement activity - Ribonuclease H (RNase H) - Blocker (a short chain of single stranded oligonucleotides)	47–56	30–60	Table S8
HDA	2	- Helicase - *Bst* DNA polymerase - SSB protein	37–65	30–120	Table S8
CPA	5	- *Bst* DNA polymerase	63	60	Table S8

* NR, not required.

### 2.1 Thermo-cycle amplification techniques

#### 2.1.1 Conventional PCR

**Fig. 1:**
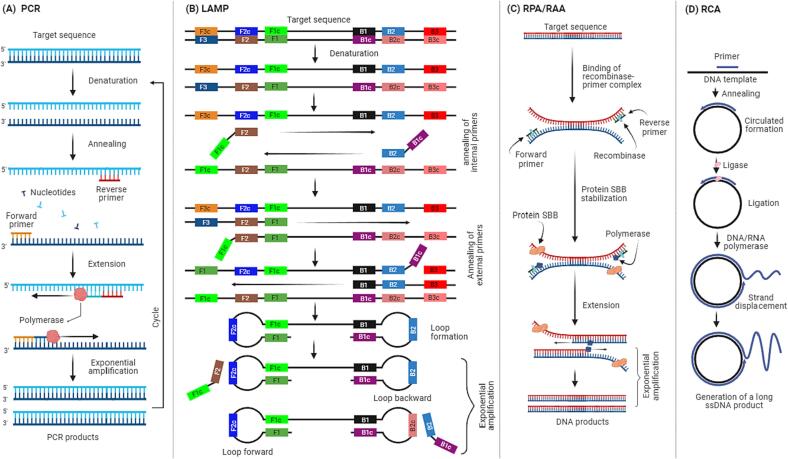
A schematic representation of the PCR, NASBA, RPA/RAA, and RCA technique.

Fig. 1A presents the schematic mechanism of the PCR technique, a process involving three main steps in each cycle: denaturation, where the DNA strands separate at high temperature; annealing, where short DNA primers attach to target sequences at lower temperature; and extension, where DNA polymerase adds nucleotides to the primers at a moderate temperature (Kabiraz et al., 2023a). This cycle is typically repeated around 20–40 times. With each cycle, the target DNA segment doubles in quantity, resulting in exponential amplification. Conventional PCR has been used to detect various pathogens in a variety of food samples, including seafood, milk, rice cake, eggs, and vegetables in recent studies (Table S1). The limit of detection (LOD) of this method varies among the types of food and pathogen species. For example, Fang et al. (2021) reported that conventional PCR could detect *V. cholerae* in shrimp at a concentration of 10^3^ cfu/mL, whereas Liu et al. (2022) observed that this method could detect *S. Pullorum* in eggs at a concentration of 10^5^ cfu/mL. Sun et al. (2022) reported that the detection limit for *L. monocytogenes* in milk samples was approximately 10^4^ cfu/mL after enriching the samples for 9 h. Furthermore, recent studies have also been constantly developing approaches to detect multiple pathogens in a single test tube. For instance, Hernández et al. (2022) conducted a study to simultaneously detect *Salmonella* spp., *Shigella* spp., *E. coli*, and norovirus in lettuce, coriander, strawberry, or raspberry using a single-tube reaction. The authors reported that the detection limit for norovirus was 1–100 pfu/mL without the need for sample enrichment, while for *Salmonella* spp., *Shigella* spp., and *E. coli* it was 1–10 cfu/mL after 24 h of enrichment. Similarly, Liu et al. (2022b) reported the simultaneous detection of *Salmonella* Pullorum and *Salmonella* Enteritidis in chicken eggs with a concentration of 10^4^ cfu/mL after enriching the samples for 2 h; they further demonstrated that enriching the chicken egg samples for 6 h and 10–12 h could enable the detection of these pathogens at a concentration of 10 and 1 cfu/mL, respectively.

While enrichment can enhance the sensitivity of pathogen detection, it is not always ideal for achieving timely results. Thus, recent studies have developed innovative approaches to improving the performance of conventional PCR in detecting pathogens in food samples by combining PCR array with other essential approaches, such as new electrophoresis-based separation techniques, nanomaterial-based, and CRISPR/Cas-based technologies. These methods eliminate the need for sample enrichment entirely or, at the very least, significantly reduce the required enrichment time. For example, He et al. (2022) combined PCR with the use of magnetic nano carbon dots (Mag-Cds) and an electrokinetic separation technique, the so-called capillary electrophoresis (CE), to improve the detection of pathogens in food samples. In their study, the Mag-Cds were used to capture the pathogen bacteria cells before DNA extraction, whereas the CE was used to allow the amplicon analysis. The authors reported that their method could simultaneously detect the presence of *S. aureus*, *E. coli* O157:H7, *Salmonella* Typhimurium, and *L. monocytogenes* in milk samples at a concentration of 10 cfu/mL without the enrichment of samples. They also highlighted that their developed method was 6 times faster than the cultured-based methods. Furthermore, a recent study conducted by Campàs et al. (2023) employed a PCR array coupled with an electrochemical biosensor to detect pathogens in food samples. Although they reported that their method could detect the presence of 1 cfu/mL of *V. parahaemolyticus* in oysters, their samples required 6–24 h of enrichment before analysis.

The detection of foodborne pathogens in food samples using conventional PCR has also been combined with CRISPR/Cas-based technology to improve detection specificity and sensitivity; this technology refers to a revolutionary gene-editing system that utilizes the clustered regularly-interspaced short palindromic repeats (CRISPR) and CRISPR-associated (Cas) proteins. In this approach, the amplified product from PCR is subjected to CRISPR/Cas-based analysis, enabling the identification of the target pathogen. Zhang et al. (2020) employed a PCR array coupled with CRISPR/Cas-based technology to enable the detection of a pathogen in seafood samples. Their method used Cas12a to perform dsDNA cleavage under the guidance of the CRISPR ribonucleic acid (crRNA). By coupling the PCR array with CRISPR/Cas-based technique, they could detect *V*. *parahaemolyticus* at a concentration of 100 cfu/mL in shrimps without sample enrichment. Interestingly, the authors mentioned that their developed system could potentially be used in on-site detection as it provides an endpoint visualization that can be observed by the naked eye.

#### 2.1.2 Real-time PCR

Real-time PCR (also known as “qPCR”) measures the amplification of DNA during each PCR cycle using fluorescent dyes or probes (Kabiraz et al., 2023a). In qPCR, the fluorescence signal increases as the amplified DNA accumulates. The number of cycles needed for the fluorescence to cross a certain threshold (Ct) is used to quantify the initial amount of DNA template. In recent studies, real-time PCR (qPCR) has been used to detect and simultaneously quantify the pathogens in various types of food, including eggs, meat, seafood, and fresh vegetables (Table S2). Similar to conventional PCR, the sensitivity of qPCR varies between pathogen species, food types, and enrichment time. Kim et al. (2020) reported that the qPCR method could detect the presence of *E. coli* O157:H7 in lettuce and beef at a concentration of 10^3^ cfu/mL. In the same study, using the same type of food samples, the authors also observed that this method was able to detect the presence of *L. monocytogenes* and *Salmonella* Typhimurium at a concentration of 10^2^ cfu/mL. Kim et al. (2021) reported similar results by using qPCR for detecting the presence of *S. aureus*, *S. capitis*, *S. caprae*, and *S. epidermidis* in milk. The authors found that the detection limit for these four *Staphylococcus* species was approximately 10^2^ cfu/mL. However, this method was only capable of detecting the presence of *Campylobacter* spp. in chicken samples at a concentration of 3.2 × 10^5^ cfu/mL, even after enriching the samples for 24–48 h. Nevertheless, this method could detect the presence of *L. monocytogenes, Salmonella* spp.*,* and *E. coli* in pork, beef, chicken, and mung bean sprout at a concentration of 1 cfu/25 g after enriching the samples for 18 h (Bundidamorn et al., 2021). In another study, Garrido-Maestu et al. (2020) also reported that they could detect the presence of *E. coli* O157 in ground beef and leafy greens at concentrations of 3.9 cfu/25 g and 3.3 cfu/25 g, respectively, after enriching their samples for 3 h.

To enhance the detection performance of qPCR, researchers have primarily focused on improving the sample pre-treatment and preparation, which involve bacterial isolation, bacterial concentration, and DNA concentration. An example of this is the work of Park et al. (2020) which combined qPCR with immunomagnetic separation (IMS-qPCR) to detect *E. coli* O157:H7 in different types of food samples. They used IMS to improve the isolation of bacterial cells from the complex food matrix using immunomagnetic beads. By employing this approach, the authors achieved successful detection of *E. coli* O157:H7 in milk, ground beef, and cabbage samples at concentrations ranging from 10 to 10^2^ cfu/mL. Interestingly, their method required a short enrichment time of only 1 h, and the entire assay was completed within 3 h. Similarly, another noteworthy study by Lee et al. (2022b), who also employed IMS-qPCR detect *E. coli* O157:H7 and *Salmonella* Typhimurium in fresh-cut apples, reported a successful detection of these pathogens at concentrations of 2.7 × 10 cfu/mL and 1.8 × 10^2^ cfu/mL, respectively, without the need for sample enrichment. However, it's important to note that IMS-qPCR's performance may vary depending on the specific food sample being analyzed. For example, Dhital & Mustapha (2023) employed a similar strategy and found that the detection limit for *E. coli* O157:H7 in chicken breast, ground beef, tomato, and romaine lettuce was as low as 10 cfu/mL, albeit with a longer enrichment time ranging from 4 to 8 h.

DNA concentration is another approach that could improve the qPCR performance. Generally, DNA concentration involves eluting the purified DNA in a limited amount of elution buffer. In a recent study, Kim et al. (2020) reported that combining qPCR with DNA concentration could detect *E. coli* O157:H7, *L. monocytogenes*, and *Salmonella* Typhimurium in lettuce samples at concentrations as low as 10^2^ cfu/mL without the need for sample enrichment. Similar results were observed when this method was applied to detect the presence of *C. sakazakii* in rice cereal and powdered formula milk (PIF) samples (Xie & Liu, 2021), and *Salmonella* Typhimurium, *S. aureus*, and *L. monocytogenes* in milk samples (Shi et al., 2021). Interestingly, another study reported that this method could detect the presence of *E. coli* O157:H7 in chicken breast and romaine lettuce samples as well as the presence of *S. aureus* in rice cereal and PIF samples at a concentration of 10 cfu/mL (Dhital and Mustapha, 2023, Xie and Liu, 2021). However, the detection of 10 cfu/mL of *E. coli* O157:H7 in ground beef, ground turkey, and tomato samples using this method requires 4 h of sample enrichment (Dhital & Mustapha, 2023). Remarkably, a lower detection limited was obtained when qPCR was combined with a sample filtration process and DNA concentration (Kim et al., 2020, Kim and Oh, 2020a, Kim and Oh, 2021). In this method, bacterial cells were concentrated using a filtration membrane before extracting the DNA; it reported the success detection of the presence of 1 cfu/25 g of *E. coli* O157:H7 and *Salmonella* Typhimurium in lettuce and cabbage samples (Kim & Oh, 2020a), as well as 10 cfu/mL of *E. coli* O157:H7, *L. monocytogenes*, and *Salmonella* Typhimurium in lettuce and beef samples (Kim et al., 2020), without the need for sample enrichment. In another study, this method detected the presence of *E. coli* O157:H7 in cabbage samples at a concentration of ≤ 7 cfu/25 g, although the samples required undergoing a 2 h enrichment process (Kim & Oh, 2021).

Moreover, sample treatment with bacteriophage could also improve the performance of qPCR in detecting pathogens in food samples and enable the detection of viable cells. For example, Huang et al. (2023) employed qPCR coupled with dual-phage amplification techniques using bacteriophage SEP37 and reported that this approach could detect the presence of viable *S. enterica* and *S. aureus* in milk and lettuce samples at a concentration of 10 cfu/mL without the need for sample enrichment. Although this method offers rapid and sensitive detection of pathogens, the application of this approach has not yet become popular in food testing (Jones et al., 2020). Future studies are therefore suggested to explore the application of this technique in a broad range of pathogens in various types of food.

#### 2.1.3 Digital PCR

In digital PCR, the DNA sample is partitioned into numerous individual reactions and each partition is analyzed separately for target DNA amplification (He et al., 2020, Lei et al., 2020). This process involves three steps in each partition: partitioning the sample, performing PCR within each partition, and analyzing the results. By using a limiting dilution approach, the presence or absence of target DNA is determined based on the number of positive partitions. This technique enables absolute quantification of target DNA molecules in the original sample, offering higher sensitivity and precision compared to traditional quantitative PCR methods. Unlike traditional qPCR, which relies on the measurements of Ct, digital PCR uses Poisson statistical analysis to estimate the initial concentration of target pathogens. Digital PCR could exhibit a remarkable ability in detecting low-level targets and be more resistant to PCR inhibitors. Recent studies demonstrated that this method could be used to detect a low number of pathogens in seafood and fruit juice (He et al., 2020, Lei et al., 2020). Lei et al. (2020) reported that this method could detect the presence of *V. parahaemolyticus* in clams at a concentration of 15 cfu/mL. In a separate study, He et al. (2020) used this technology to detect the presence of enterohaemorrhagic *E. coli* O157:H7 in apple juice. The authors reported that they were able to detect this pathogen in their samples at a concentration of 2 cfu/mL.

### 2.2 Isothermal amplification techniques

#### 2.2.1 Loop-mediated isothermal amplification (LAMP)

Fig. 1B presents the schematic representation of the LAMP amplification. The LAMP primer set includes internal primers (FIP and BIP) as well as external primers (F3 and B3) (Du et al., 2022, Jia et al., 2023, Jiang et al., 2020, Li et al., 2021, Priya et al., 2020, Wang et al., 2020a, Xie et al., 2022a, Xiong et al., 2020, Zendrini et al., 2021, Zhang et al., 2021, Zhang et al., 2022). FIP comprises the F1c and F2 regions, while BIP comprises the B1c and B2 regions. The FIP primer, located upstream, contains the F2 region, which complements the F2c region at the 3′ end of the target gene and shares the same sequence as the F1c region at the 5′ end of the target gene. The F3 primer, an upstream external primer, encompasses the F3 region, which matches the F3c region of the target gene. On the other hand, the BIP primer, positioned downstream, comprises the B2 region, complementing the B2c region at the 3′ end of the target gene and sharing an identical sequence with the B1c region at the 5′ end of the target gene. Lastly, the B3 primer, a downstream external primer, contains the B3 region, which is complementary to the B3c region of the target gene. The amplification by this technique requires a constant temperature ranging between 60 and 65 °C. Typically, the amplification can be completed within 30–60 min.

In recent years, a great number of studies have been devoted to exploring the capability of LAMP technology in detecting various pathogens in various types of foods, including milk, seafood, and meats (Du et al., 2022, Jia et al., 2023, Jiang et al., 2020, Li et al., 2021, Priya et al., 2020, Wang et al., 2020a, Xie et al., 2022a, Xiong et al., 2020, Zendrini et al., 2021, Zhang et al., 2021, Zhang et al., 2022). Most of these studies used fluorescence to indicate the presence of pathogens in the amplified product and some of them applied necessary pre-treatment on their samples before DNA amplification (Table S3). Interestingly, several of these studies reported that LAMP technology enabled direct recognition of signals with the naked eye, which is probably suitable for on-site detection. For example, the presence of *Salmonella* or *Campylobacter* in chicken meat could be detected by observing the turbidity anomaly or colorimetric changes during the amplification process (Wang et al., 2020a, Zendrini et al., 2021). Similarly, Li et al. (2021) also reported that this method enabled the detection of *Salmonella* in pork by simply observing the colorimetric changes with the naked eye. These studies demonstrated that LAMP technology could allow the detection of low concentrations of pathogens in food samples; thus, enrichment may not be necessary. For example, the presence of *Salmonella* Typhimurium in chevron and chicken meat could be detected at concentrations of 8.5 cfu/g and 55 cfu/mL, respectively, without the need for enrichment (Jia et al., 2023, Priya et al., 2020).

Strikingly, the use of the LAMP method could yield a comparable result to PCR and sometimes even better. For example, Xiong et al. (2020) reported that the LAMP method was 10 times more sensitive than that of conventional PCR in detecting the presence of *S. aureus* in fish samples. In a separate study, Zendrini et al. (2021) reported that both LAMP and PCR methods were able to detect the presence of *Salmonella* and *Campylobacter* in chicken meat down to 10 cfu/g and 10^3^ cfu/g, respectively, without enriching their samples. It is worth mentioning that a study mentioned that while LAMP technology did not show better performance than PCR-based methods, it could double down the cost of operation (Xie et al., 2022a).

To enhance the usability of LAMP as a field-testing tool, several researchers have investigated the integration of this technology with other diagnostic methods, such as lateral flow assay (LFA) and aptamers. In a recent study, Jiang et al. (2020) employed LAMP technology combined with LFA to simultaneously detect *Salmonella* spp., *Cronobacter* spp., and *S. aureus* in PIF and found that these pathogens could be detected at concentrations as low as 4.2, 2.6, and 3.4 cfu/g, respectively, without the need for enrichment (Jiang et al., 2020). In another study, Jia et al. (2023) utilized LAMP technology combined with aptamers and RNase H2 enzyme for the detection of *Salmonella* Typhimurium in chicken meat samples. The specific aptamers were used to capture bacterial cells, whereas RNase H2 enzyme was used to enable visual detection. Remarkably, this approach facilitated the successful detection of the pathogen in chicken samples at a concentration as low as 5.5 cfu/mL (100 times lower compared to conventional LAMP methods) without the need for sample enrichment (Jia et al., 2023). These findings indicate that LAMP has the potential to serve as an alternative to PCR-based methods and potentially can be utilized as an on-site detection tool.

#### 2.2.2 Recombinase polymerase amplification (RPA)/recombinase aided amplification (RAA)

Fig. 1C presents the schematic representation of the RPA amplification, a technique utilizing recombinase and polymerase enzymes to amplify target nucleic acid. The RPA process involves the formation of recombinase-primer complexes that scan the DNA template for homologous sequences and create D-loop structures, followed by the binding of the polymerase enzyme to extend the 3′ end of the primer, leading to exponential amplification (Cai et al., 2021, Chen et al., 2021, Ma et al., 2020, Wang et al., 2021, Wang et al., 2020b). This technique can produce millions of target copies at low temperatures (37–42 °C) in less than 1 h, with a detection limit as low as a single target copy. Regarding the RAA, this technique utilizes a similar mechanism to RPA. The only discernible difference between RPA and RAA is likely the source of the polymerase used, with RPA using Phage T4 polymerase while RAA utilizes bacterial or fungal recombinases.

In recent studies, researchers have used RPA and combined it with LFA systems, fluorescent dyes, fluorescent probes, or CRISPR/Cas-based technology to detect pathogens in food samples (Cai et al., 2021, Chen et al., 2021, Jin et al., 2022, Liu et al., 2022a, Ma et al., 2020, Mao et al., 2022, Petrucci et al., 2022, Tian et al., 2021, Wang et al., 2021, Wang et al., 2022b) (Table S4). For example, Ma et al. (2020) utilized RPA in combination with a lateral flow dipstick to identify various pathogens in different types of food samples. The authors reported successful detection of *Staphylococcus aureus*, *Vibrio parahaemolyticus*, and *Salmonella enteritidis* in various types of seafood at concentrations of approximately 41, 80, and 26 cfu/mL, respectively. Wang et al. (2021) used a similar approach to detect *V. cholerae* and *V. vulnificus* in shrimps and found that these pathogens could be detected at a concentration of 1 cfu/10 g after 4 h of enrichment. This approach has also been used by Petrucci et al. (2022) to detect *E. coli* O157:H7 in chicken meat and they found that this pathogen could be detected at a concentration of 10 cfu/mL after enriching their samples for 10 min.

An interesting study presented by Chen et al. (2021) evaluated the effectiveness of the material used in their lateral flow systems. In their study, they evaluated the use of RPA combined with common LFA (colloidal gold-based LFA) and europium nanoparticles-based LFA (EuNP-based LFA) to detect various types of pathogens in a variety of types of food samples. Their study showed that using RPA coupled with common LFA could detect *L. monocytogenes*, *V. parahaemolyticus*, *E. coli* O157:H7 in beef, milk, chicken breast, and shrimp samples at a concentration of 90, 70, and 40 cfu/mL, respectively, without the need of enrichment. When they used the RPA coupled with EuNP-based LFA, they could detect these pathogens in these food samples at concentrations of 9, 7, and 4 cfu/mL (10 times more sensitive that of common LFA), respectively, also without the need for enrichment (Chen et al., 2021). In a separate study, Jin et al. (2022) reported that the RPA array coupled with a gold nanoparticle-based LFA (AuNP-based LFA) could detect *V. parahaemolyticus*, *S. aureus*, *S. enterica*, *E. coli* O157:H7, and *L. monocytogenes* in chicken, pork, beef, milk, shrimp, and fish samples simultaneously, with a recovery rate of more than 90%.

A combination of RPA arrays with CRISPR/Cas12a (RPA-CRISPR/Cas12a) can perform better than or at least comparable to the qPCR method in detecting pathogens in food samples (Liu et al., 2022a, Tian et al., 2021, Xiao et al., 2022). For example, Tian et al. (2021) compared the use of RPA-CRISPR/Cas12a and qPCR in detecting *L. monocytogenes* in milk and found that the sensitivity of RPA-CRISPR/Cas12a was 100 times higher than that of the qPCR method, with a detection limit of 10 CFU/mL, without requiring enrichment. Similarly, Xiao et al. (2022) found that RPA-CRISPR/Cas12a outperformed the qPCR method in detecting *Y. enterocolitica* in pork, as it was able to detect this pathogen at a concentration of 1 cfu/mL, whereas qPCR could only detect it at a concentration of 10^2^ cfu/mL. In a separate study, Liu et al. (2022a) compared the specificity and sensitivity of RPA-CRISPR/Cas12a and qPCR in detecting *Salmonella* spp. in egg samples and found that RPA-CRISPR/Cas12a was similar to qPCR without the need for sample enrichment. However, when the authors enriched the sample for 3 h, they found that RPA-CRISPR/Cas12a was more sensitive than qPCR (Liu et al., 2022a).

Regarding the RAA, this method has also been proven to be able to detect pathogens in various food samples, such as shrimps, clams, fish, milk, and chicken meats (Table S5). For example, RAA was reported to be able to detect *E. coli* O157:H7 in milk samples without the need for enrichment, at a concentration ranging from 8 to 54 cfu/mL (Mu et al., 2021, Mu et al., 2022). Further investigation showed that this method could detect *E. coli* O157:H7 in lettuce at a concentration of 70 cfu/mL and *Salmonella* Typhimurium in chicken meat at a concentration of 10 cfu/mL without the need for enrichment (Mu et al., 2021, Wang et al., 2022b). In recent studies, there has been a focus on enhancing the performance of RAA technology and aiming for its utilization as an on-site testing tool. For instance, Fang et al. (2021) combined RAA with LFA (RAA-LFA) and found that their method could detect *V. cholerae* in shrimp samples at a concentration as low as 46 cfu/mL within 50 min of the assay. When the authors compared the sensitivity of this technology to conventional PCR, they found that RAA-LFA was ten times more sensitive than the conventional PCR method. However, another study by Li et al. (2023) observed that the food matrix affected the sensitivity of RAA-LFA. In their study, they found that the detection limit for *V. parahaemolyticus* in shrimp and clam was 7.4 × 10^4^ CFU/g, 10 times higher than that of fish (Li et al., 2023). The authors speculated that fish matrices contain more RAA inhibitors than the shrimp and clam (Li et al., 2023). Furthermore, the combination of RAA with CRISPR/Cas-based technology (RAA-CRISPR/Cas12a) has demonstrated its capability to detect *C. jejuni* in chicken meat samples at concentrations ranging from 0.12 to 1.2 cfu/mL (Zhi et al., 2022). Despite achieving successful detection of low concentrations of *C. jejuni*, the requirement for sample enrichment for 24 to 48 h poses a drawback to this method (Zhi et al., 2022). However, a comparison with the traditional culture-based method revealed that RAA-CRISPR/Cas12a outperformed it by tenfold in detecting *C. jejuni* in chicken meat samples (Zhi et al., 2022). Taken together, these findings indicated that RAA technology is a great alternative to culture- or PCR-based methods.

#### 2.2.3 Rolling circle amplification (RCA)

Fig. 1D presents the schematic representation of the RCA method. The RCA process involves the utilization of DNA/RNA polymerase (such as phi29 DNA polymerase or T7 RNA polymerase), a short linear single-stranded DNA or RNA primer, a circular template, and ligase (Guo et al., 2022a, Liu et al., 2020, Liu et al., 2021, Prasad et al., 2023, Xu et al., 2021, Yuan et al., 2022, Zhan et al., 2020). This combination of components leads to the generation of a lengthy single-strand product through DNA/RNA polymerase action, resulting in a single-strand RCA product (RCAP). This RCAP is complementary to the circular template used. Furthermore, specific oligonucleotide padlock probes (PLP) and either T4 DNA ligase or a specialized single-stranded DNA ligase collectively facilitate the transformation of bacterial single- or double-strand RNA/DNA templates into single-strand circular DNA.

In recent studies, RCA technology has been used to detect numerous pathogens (e.g., *Cronobacter* spp., *L. monocytogenes*, *C. sakazakii*, *C. perfringens*, *Salmonella* spp*.*, and *V. parahaemolyticus*) in various food samples including milk, kalakhand, lettuce, PIF, pork, fruit juice, and oyster (Table S6). Note that most of the recent studies enabled detection by using optical sensing through fluorescent signals from fluorescent dyes or probes. When RCA was used to detect *Cronobacter* spp*.* and *L. monocytogenes* in milk samples, this technology could detect these pathogens at concentrations of 4.5 × 10^2^ cfu/mL and 4.4 × 10^2^ cfu/mL, respectively, without the need for enrichment (Liu et al., 2020, Prasad et al., 2023). In a separate study, Guo et al. (2022) reported that this technology could detect *Salmonella* Typhimurium and *S. flexneri* in milk samples at a concentration of 10 cfu/mL without enrichment. Interestingly, the authors reported that the RCA assay was 100 times more sensitive than the qPCR method in detecting these pathogens in their tested samples (Guo et al., 2022a). In a separate study, Prasad et al. (2023) reported that the RCA array could detect *L. monocytogenes* in milk and kakahand at concentrations of 4.4 × 10^2^ cfu/mL and 9.4 × 10^2^ cfu/mL, respectively, without sample enrichment and 4.4 cfu/mL in milk and 9.4 cfu/mL after 3–6 h of enrichment. The authors also noted that this method was 100 times more sensitive than conventional PCR in detecting *L. monocytogenes* in their tested food samples.

Recent studies have also attempted to integrate RCA with biosensors. However, this approach does not seem able to detect the presence of pathogens in food samples at low concentrations as reported in recent studies. For instance, coupling RCA coupled with an aptasensor could only detect *L. monocytogenes* in lettuce at a concentration of 6.1 × 10^3^ cfu/mL and *C. sakazakii* in PIF at a concentration of 2.4 × 10^3^ cfu/mL (Liu et al., 2021, Zhan et al., 2020). Therefore, more effort is needed to improve the performance of RCA in conjunction with biosensors in detecting pathogens in food samples. Nevertheless, a study reported that RCA technology could detect pathogens in pork better than conventional PCR and qPCR (Milton et al., 2021b).

#### 2.2.4 Saltatory rolling circle amplification (SRCA)

**Fig. 2:**
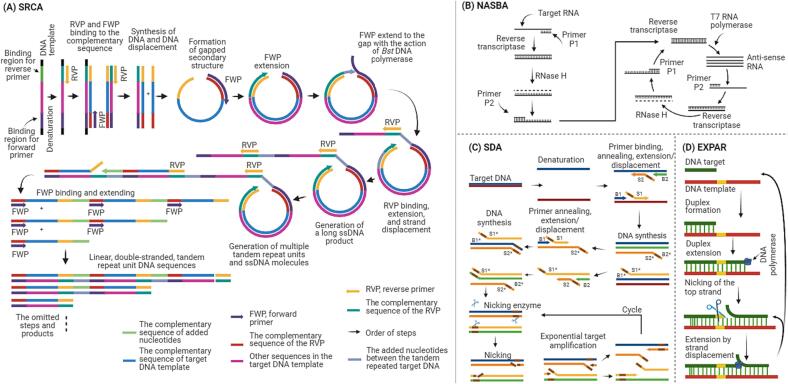
A schematic representation of the SRCA, NASBA, SDA, and EXPAR technique.

Fig. 2A presents the schematic representation of the SRCA method. SRCA follows an initial amplification mechanism similar to RCA, using a circular DNA template and a DNA polymerase with strand displacement activity. However, during the SRCA process, specific recognition sites within the template sequence are encountered (Milton et al., 2021b, Milton et al., 2021a, Milton et al., 2021c, Zhang et al., 2019a). At these recognition sites, DNA-cutting enzymes, such as restriction endonucleases or nicking enzymes, introduce breaks in the circular DNA. These breaks create free 3′ ends that serve as priming sites for the initiation of new DNA synthesis. As a result, SRCA produces shorter, non-contiguous DNA segments, referred to as saltatory products. In recent studies, this method has been used to detect numerous pathogens in various types of food samples, including PIF, milk, pork, chicken meat, and oysters (Huang et al., 2023b, Milton et al., 2021b, Milton et al., 2021a, Milton et al., 2021c, Prasad et al., 2023, Yang et al., 2019, Yuan et al., 2022, Zhang et al., 2019a).

Remarkably, researchers have reported that the SRCA method outperforms the PCR method in detecting pathogens in food samples (Milton et al., 2021b, Milton et al., 2021a, Milton et al., 2021c, Zhang et al., 2019a). For example, the SRCA method demonstrated a detection limit of 340 cfu/mL for *C. sakazakii* and 560 cfu/mL for *S. aureus* in PIF, without the need for sample enrichment (Yang et al., 2019, Zhang et al., 2019c). These studies noted that SRCA was 100-fold more sensitive than the results achieved by conventional PCR (Yang et al., 2019, Zhang et al., 2019c). Other studies reported that SRCA could detect 10^3^ cfu/g of *C. jejuni* in chicken (Milton et al., 2021a), 40 cfu/g of *S. enterica* in pork (Milton et al., 2021b), and 80 cfu/g of *C. perfringens* in pork (Milton et al., 2021c), all without the need for enrichment. In evaluations involving these pathogens, researchers found that SRCA demonstrated 10- and 100-fold higher sensitivity compared to conventional PCR and qPCR, respectively (Milton et al., 2021b, Milton et al., 2021a, Milton et al., 2021c, Milton et al., 2021c). Guo et al. (2022a) reported that employing SRCA could simultaneously detect *Salmonella* spp. and *Shigella* spp. in skimmed milk at a concentration as low as 10 cfu/mL without sample enrichment, which was 100-fold lower than that of multiplex qPCR. In a separate study, Guo et al. (2022b) used the same approach and observed that they could detect *S. aureus* in spiked pork at a concentration of 66 cfu/g through gel electrophoresis in milk samples. Prasad et al. (2023) reported that the detection limit for *L. monocytogenes* in milk and kalakhand was 440 cfu/mL and 940 cfu/mL, respectively, without the need for enrichment. With the exclusion of te enrichment step, the authors observed that the utilization of SRCA resulted in detection sensitivities 100- and 10-fold greater than those achieved by conventional PCR when detecting *L. monocytogenes* in milk and kalakhand, respectively.

#### 2.2.5 Nucleic acid sequence-based amplification (NASBA)

Fig. 2B presents the schematic representation of the NASBA amplification. This method targets 16 s rRNA genes or messenger (m)RNA transcripts to detect bacteria, enabling the analysis of bacterial viability. The NASBA process involves the amplification of single-strand RNA using two primers and three enzymes: avian myeloblastosis virus reverse transcriptase (AMV-RT), RNase H, and T7 DNA-dependent RNA polymerase (DdRp) (Kumar, 2021, Zhai et al., 2019). In this technique, AMV-RT extends the primers to generate complementary DNA, and RNase H forms double-stranded DNA. The T7 DdRp recognizes the exposed T7 promoter of the double-strand DNA and initiates transcription, thus commencing the reaction. Since NASBA enzymes are heat labile, the amplifications can be carried out at relatively low temperatures, with optimal conditions set at 41 °C for 1.5–2 h. Following amplification, the products can be detected using various methods, including gel electrophoresis, enzyme-linked immunosorbent assay (ELISA), enzyme-linked gel assay, electrochemiluminescent (ECL) techniques, and real-time monitoring with molecular beacons.

In recent years, the application of the NASBA technique to detect foodborne pathogens in food samples has been demonstrated by considering the presence of background microbiota, which is important to accurately portray the actual state of food samples. For instance, Zhai et al. (2019) reported detecting *Salmonella* in pork at concentrations as low as 9.5 × 10^3^ cfu/mL using a real-time NASBA technique, following 12-h enrichment of their samples with pork background microbiota. In a subsequent study, Zhai et al. (2022) introduced a duplex real-time NASBA with a molecular beacon approach to simultaneously identify viable cells of *Salmonella* spp. and serotype Paratyphi C in pork and chicken samples. Notably, their method detected these pathogens and their serovars in tested food samples at concentrations as low as 5 cfu/25 g, following 12-h sample enrichment. Furthermore, the NASBA technique can be synergistically combined with other essential methods, such as the CRISPR/Cas-based system, to enhance pathogen detection capabilities. In a recent investigation, Xue et al. (2022) demonstrated that the integration of NASBA with the CRISPR/Cas13-based system facilitated the detection of *Salmonella* at concentrations as low as 1.5 cfu/mL in pure cultures. In their method, the CRISPR/Cas13a was used to serve as the reporter of NASBA. However, there remains a scarcity of information regarding the utilization of this innovative approach to detect foodborne pathogens within food samples, specifically considering the presence of background microbiota.

#### 2.2.6 Strand displacement amplification (SDA)

Fig. 2C illustrates the schematic mechanism of SDA. This method employs DNA polymerases with strand displacement activity and operates isothermally at a constant temperature range of 37 °C to 65 °C. The SDA process commences with the denaturation of the target dsDNA at an elevated temperature, typically around 90 °C, enabling the binding of primers (B1, B2, S1, and S2) (Cai et al., 2019). Notably, primers S1 and S2 contain HincII recognition sites. Following primer binding, *exo*-klenow facilitates their simultaneous extension in the presence of nucleotides. Subsequent to extension, primers B1 and B2 displace the extension products of S1 and S2, designated as S1* and S2*. This displacement is pivotal, enabling the remaining primers (B1, B2) to bind to S1* and S2*, followed by another round of *exo*-klenow-mediated extension. This extension yields extended primers with two phosphorothioate HincII recognition sites and products featuring a single phosphorothioate HincII recognition site. HincII cleaves the amplified target sequences at the nick and further extension ensues, allowing for iterative cycles of amplification.

In recent years, SDA has been used in combination with other essential techniques to detect the presence of pathogens in food samples. For example, Cai et al. (2020) employed a combined approach involving the SDA method, an aptamer, and molecular beacons to detect *S. aureus* in broth and milk samples. Within their study, the aptamer functioned as a nucleic acid-based receptor molecule, exhibiting targeted binding to the pathogen of interest, thereby facilitating accurate and effective recognition. Notably, while their established technique effectively detected the pathogen within a pure culture at a concentration of 1.7 cfu/mL, their findings indicated limited sensitivity when applied to milk samples. Specifically, their method enabled detection in milk only at concentrations as low as 1 × 10^4^ cfu/mL with a 95% recovery rate. Nevertheless, they reported that their approach could achieve amplification within just 45 min, representing a significant reduction in time when compared to the original 2-h SDA process, which has been deemed overly lengthy for the development of biosensors.

#### 2.2.7 Exponential amplification reaction (EXPAR)

Fig. 2D presents the schematic representation of the EXPAR amplification. The process of EXPAR begins when the target sequence primes to the trigger sequence on the template, generating a partial double-strand duplex (Xia et al., 2022, Xu et al., 2022). Subsequently, DNA polymerase extends this duplex, yielding an extended double-strand DNA segment that incorporates a recognition site for a nicking enzyme. The subsequent step involves the action of the nicking enzyme, which cleaves the upper strand of the DNA, triggering the DNA polymerase to perform strand displacement, leading to the displacement of the cleaved trigger sequence. As a result of this displacement, additional trigger sequences are generated. Importantly, this sequence of events recurs in a repetitive and exponential manner, further contributing to the amplification process. Thus far, the application of this method for detecting pathogens in food samples has been limited. Nonetheless, in a recent study, Xu et al. (2022) coupled the EXPAR technique with an immune sandwich structure comprising antibodies and aptamers. This coupling enhanced the isolation of *C. sakazakii* from milk samples, enabling the detection of this pathogen even at concentrations as low as 12 cfu/g. The authors highlighted that their approach facilitated the detection of this microorganism in the tested samples in under 2 h.

#### 2.2.8 Single primer isothermal amplification (SPIA)

**Fig. 3:**
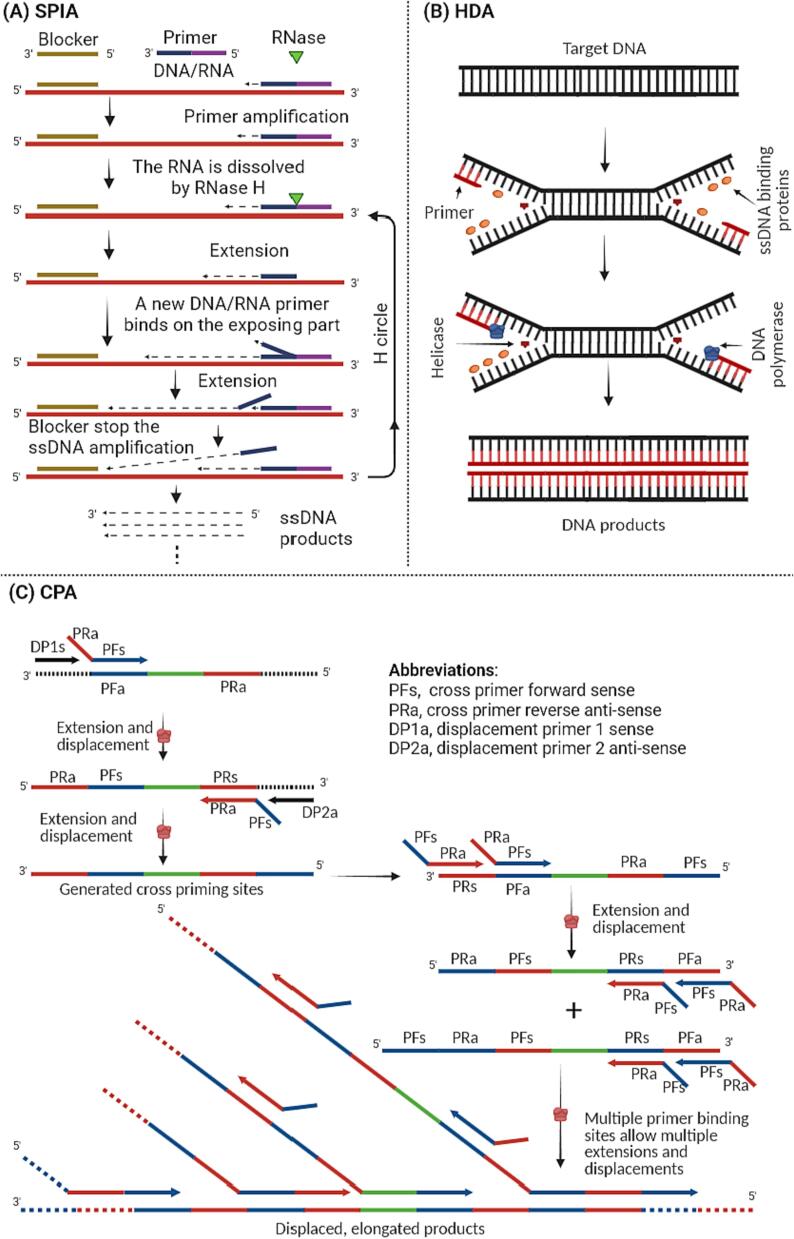
A schematic representation of the SPIA, HDA, and CPA technique.

Fig. 3A presents the schematic representation of the SPIA method. The mechanism of this method involves the use of a single, target-specific chimeric primer with a 3′-DNA sequence portion and a 5′-RNA sequence portion (Yang et al., 2020a, 2021; Yin et al., 2022). The amplification system includes a DNA polymerase with robust strand-displacement activity, RNase H, and a blocking oligonucleotide. The process initiates with the chimeric primer binding to the complementary sequence in the target DNA, followed by extension using the DNA polymerase. Once the DNA amplification is completed, the RNA portion in the chimeric primer is cleaved by RNase H, revealing the primer-binding site for further rounds of primer annealing and extension. Simultaneously, the DNA polymerase displaces the previous product, generating single-strand DNA (ssDNA), a cyclic process involving primer binding, extension, displacement, and cleavage, resulting in the efficient generation of multiple amplification products. Typically, the whole amplification process can be completed within 30–60 min (Yang et al., 2020a, Yang et al., 2021).

In a recent study, Yang et al. (2020) developed SPIA combined with a fluorescent dye to detect *L. monocytogenes* in raw chicken in real-time. The author reported that their method could detect this pathogen in their tested samples at concentrations as low as 1.4 cfu/g. Notably, they underscored the remarkable sensitivity of their SPIA method, which was 100-fold greater than that achieved by the conventional PCR approach. In a separate study, Yang et al. (2021) used a similar method and reported that they could detect *V. parahaemolyticus* in raw oysters at a concentration of 42 cfu/g. In another study, Yin et al. (2022) combined SPIA assay with an electrochemical biosensor and enabled detection of *Salmonella* in pork samples. The author observed that their method could detect this pathogen in pork at concentration as low as 68 cfu/mL without the need for enrichment. When the authors compared the detection capability of SPIA to qPCR, they found that SPIA had a higher accuracy detection than that achieved by qPCR.

#### 2.2.9 Helicase-dependent amplification (HDA)

Fig. 3B illustrates the procedural overview of the HDA technique. The process starts with thermal denaturation of the target dsDNA or RNA, typically at a high temperature around 90 °C (Lee et al., 2022a, Moon et al., 2022). The reaction temperature is then adjusted to allow primer annealing, typically within 37 °C to 65 °C. Successful primer annealing precedes the addition of a helicase enzyme, crucial for initiating the unwinding of the DNA duplex by recognizing the annealed primers, action which leads to strand separation, exposing single-strand regions ready for processing (Lee et al., 2022a, Moon et al., 2022). Following DNA duplex unwinding, DNA polymerase is introduced, triggering DNA synthesis by extending from the primers, utilizing single-strand DNA as templates. As synthesis progresses, a newly formed strand displaces the complementary strand in the target DNA duplex, yielding a fresh duplex DNA structure. This cycle repeats as helicase unwinds new duplexes, primers anneal, and DNA polymerase generates strands, achieving exponential amplification of target DNA/RNA (Lee et al., 2022a, Moon et al., 2022).

Typically, the final amplified product from HDA can be detected and analyzed using agarose gel electrophoresis and fluorescence signals obtained through a fluorescent-labeled DNA probe and SYBR gold. However, these methods necessitate expensive detectors, involve multiple experimental steps, and require extended reaction times, rendering the HDA technique impractical for field applications. In a recent study, Lee et al. (2022a) addressed this challenge by introducing the use of HRPzyme, which exhibits peroxidase-like activity when hemin is present. This innovation enables the results of the HDA to be visualized and analyzed with the naked eye. The authors demonstrated that their method enabled the observation of norovirus presence in oysters at a concentration of 10^2^ copies/mL. Nevertheless, their tested samples necessitated a 3-h enrichment period. In another study, Kim et al. (2023) reported an alternative approach, combining an HDA array with a CRISPR/Cas-based system. This strategy successfully detected *E. coli* in fresh salad mix at a concentration of 10^3^ cfu/mL without requiring enrichment steps. This advancement holds promise for rapid and sensitive pathogen detection in food samples.

#### 2.2.10 Cross priming amplification (CPA)

Fig. 3C illustrates the procedural overview of the CPA technique. In this technique, the generation cross prime sites and subsequent amplification require 5 primers and a *Bst* DNA polymerase (Xu et al., 2020, Zhang et al., 2019b). A cross primer is designed to have a non-complementary 5′-end to the template. As DNA polymerase extends an upstream displacement primer, multiple cross-linked primers (around six to eight) displace the non-complementary 5′-end of the cross primer. This displacement enables the initiation of DNA synthesis. The process leads to the exponential amplification of the target DNA sequence under a constant temperature. Regrettably, very limited information about the application of this method in detecting foodborne pathogens in food samples is available. Zhang et al. (2019b) reported that CPA could detect *Bacillus cereus* in broth at a concentration of 36 cfu/mL. In food samples, Xu et al. (2020) reported that this method could detect *E. coli* in Cantonese rice cake at concentrations ranging from 10^3^ to 10^5^ cfu/mL.

## 3 Remaining challenges and possible opportunities

### 3.1 Comparative analysis of NAA-based methods

Table 2 provides an overview of the advantages, disadvantages, and potential improvements pertaining to the NAA-based techniques. This review revealed that the application of PCR-based methods for foodborne pathogen detection in food samples is widespread, likely due to their relative sensitivity, specificity, reliability, reproducibility, rapid analysis, and wide applicability compared to traditional culture-based techniques. Thus far, PCR-based methods have been demonstrated as the most mature amplification methods that allow for simultaneous detection of multiple pathogens in a single reaction (Kim and Oh, 2020b, Wei et al., 2019). In the current state, however, PCR-based methods are unsuitable for on-site detection due to their reliance on complex sample preparation and purification as well as complex thermal cycling devices. For conventional PCR, studies showed that it can be integrated with other essential technologies such as IMS, suction filtration, CE, MCE, or CRISPR/Cas-based technology to improve sensitive and specific detection, albeit this integration necessitates expertise and specialized equipment to ensure reliable and accurate results. For qPCR, the requirement to create a reference standard curve can be time-consuming and add complexity to the quantification process. Additionally, the use of fluorescent dye-based qPCR yields relatively low specificity, formation of primer-dimer, which could lead to false positives, and limits the multiplex reaction. Employing specific probes, such as carboxyfluorescein (FAM) and Cy5, may improve the sensitivity and specificity, but requires a complex design and high cost (Bundidamorn et al., 2021). For digital PCR, this technology necessitate an adequate number of analytical droplets or chambers to facilitate Poisson distribution, as well as the requirement for appropriate sample dilution to ensure the generation of reliable data (Lei et al., 2021). Additionally, the application of digital PCR for monitoring foodborne pathogens faces challenges related to equipment and reagent costs, as well as the necessity for skilled operators (Xiang et al., 2022).

**Table 2:** Comparative analysis of the nucleic acid amplification-based methods for detecting foodborne pathogens in food samples.

Amplification method	Advantages	Disadvantages	Possible improvement
Conventional PCR	- High sensitivity and specificity - Faster than cultured based method - Able to multiplexing - Able to distinguish between the bacterial strains within the same species - Commercial kits are available abundantly - Primer design software is available offline and accessible online	- Requires thermo-cycling machine - Time-consuming as it may still require enrichment step and DNA purification - Manual operations and cumbersome peripheral equipment - Amplicon can only be analyzed after PCR process - Highly dependent on the quality of samples containing nucleic acids for amplification - Prone to PCR inhibitors - Cross contamination may occur - Lack of quantitative capacity - Optimization for multiplexing is difficult and can lead to increased cost	- Automated instrument replacing manual operations from sample preparation to detection - Design of reagents that increase the PCR’s specificity and multiplexing capability as well as reduce cross-contamination, less sensitive to PCR inhibitor - Integration with microfluidic platforms or other advanced technologies
qPCR	- High sensitivity and specificity - Faster than cultured based and conventional PCR method - Does not require post-amplification process - Allow real-time monitoring for amplification - Enable the detection and quantification simultaneously of target pathogens - High throughput due to software driven operation	- Requires thermo-cycling machine - Highly dependent on the quality of samples containing nucleic acids for amplification - High cost for instrument, reagent, fluorescent dye or fluorescent probe - Prone to PCR inhibitors - Necessity for creating standard curve - Emission spectra overlapping and nonspecific binding - Optimization for multiplexing is difficult and can lead to increased cost - Required trained personnel	- Automated instrument replacing manual operations from sample preparation to detection - Design of reagents that increase the PCR’s specificity and multiplexing capability as well as reduce cross-contamination, less sensitive to PCR inhibitor - Design of affordable and user-friendly instrument
Digital PCR	- Highly precise and accurate quantification of target molecules in complex samples - More accurate and reproducible than qPCR - Enable the detection and quantification simultaneously of target pathogens - High throughput due to software driven operation - Able to distinguish the DNA from dead and viable cells by using DNA-binding fluorescent dyes	- Requires thermo-cycling machine - High cost of instrumentation - Highly dependent on the quality of samples containing nucleic acids for amplification - Requirement for thermal cycler - Prone to PCR inhibitors - False positive results due to the presence of non-target DNA or RNA - Requires analytical droplets or chambers for the application of Poisson distribution - Complexity in designing appropriate dilution of the sample to generate accurate data - Limited multiplexing capabilities compared to qPCR - Require technical expertise	- Optimization and standardization of reaction conditions, sample preparation, and data analysis - Optimization of multiplexing capacity - Improvement of partitioning using microfluidic devices
LAMP	- Suitable for amplifying and detecting larger nucleic acids - High sensitivity and specificity - Rapid detection (less than 1 h) - More sensitive or at least comparable to conventional PCR or qPCR - High amplification efficiency - Does not require an expensive thermocycling instrument - Inexpensive instrument of amplification - Resistant to LAMP inhibitors - Support for multiplexing - Rapid and simple procedure compared with PCR methods - Cheaper than PCR-based methods - Amplification results can be accessed using fluorescent intercalating dyes or colorimetric measurement	- Requires multiple primers - primers interaction could lead to false-positive results - Formation of non-specific amplification and primer dimerization - Visual inspection or turbidity measurement of the LAMP product could lead to misjudgment - Aerosol pollution during LAMP reaction - Maintenance of the stability of LAMP reagents and primers - Tedious sample preparation	- Optimization of reaction conditions - The use of artificial intelligence (AI) for evaluating the color different in visual detection - Automated instrument replacing manual operations by integrating with microfluidic devices to reduce cross-contamination
RPA/RAA	- Rapid detection (less than 30 min) - Low-temperature requirement for amplification - The sensitivity and specificity comparable to conventional PCR/qPCR - Inexpensive instrument - Support for multiplexing - Capability of amplification without the need for DNA extraction or purification - Stable reagents at room temperature - Potential use for on-site testing - Tolerant to common amplification inhibitors - Capable of amplifying target nucleic acid from a minimal processing sample	- Requires multiple enzymes - High cost for the recombinase-primer complex - Requirement for technical expertise - Prone to non-specific amplification - Poor resolution for quantification - Affected by high DNA concentration - Stringent reaction conditions - Tedious sample preparation	- Development of the next generation of RPA/RAA technology with improved sensitivity and specificity
RCA	- High throughput detection - The sensitivity and specificity comparable to conventional PCR/qPCR - Support for multiplexing	- Highly dependent on the quality of samples containing nucleic acids for amplification - The synthesis cost is relatively high - Susceptible to background signal interference	- Design of affordable reagents that increase the RCA’s specificity and multiplexing capability
SRCA	- High specificity and high sensitivity - Sample enrichment can enhance detection sensitivity and has the potential to exceed that of conventional PCR or qPCR - Does not require padlock probe and ligase - Amplification results can be assessed visually by the presence of white precipitate or by fluorescence measurement - Simpler and cheaper than LAMP and SPIA	- Limited commercial kits for DNA purification and SRCA reactions - The necessity to select an appropriate primer pair from a substantial pool of designed primers that have been extensively validated through experimentation	- Design of affordable reagents that increase the RCA’s specificity and multiplexing capability
NASBA	- Sensitive and specific - No requirement for denaturation steps - Can directly amplify RNA fragments - DNA residues in samples will not yield a false positive signal - Potential method for the quantification of viable bacteria	- Difficulties in handling RNA - Require complex equipment for the detection of the amplified RNA products - Some food substrates inhibit the reaction - Limited by RNA secondary structure - Limited length of target sequence - Requires multiple enzymes - Enzymes are not thermostable - Less efficient in amplifying longer RNA targets - Requires a precise temperature control	- User friendly design for the amplicon analysis - User friendly application for primer design - Design of affordable reagents that increase the NASBA’s multiplexing capability
SDA	- Good sensitivity and specificity - Low equipment requirements - Simple and easy-to-control workflow	- Requires for an additional thermal denaturation for analyzing DNA - Difficult to amplify long fragment - Prone to contamination from enzymes	- Simplification of SDA procedures - Development of effective methods to reduce the contamination from the enzymes - Integration with CRISPR/Cas-based technology to improve the sensitivity and specificity detection
EXPAR	- Sensitive and specific - Combination with immunomagnetic beads and aptamer can improve the sensitivity and specificity - Rapid amplification - Does not require DNA extraction - Inexpensive instrumentation	- Complexity in designing a standard EXPAR template - Nonspecific interactions of EXPAR templates with interference DNA sequences in the sample matrix may trigger background amplification - Use high concentrations of DNA template - Not suitable for the amplification and detection of long nucleic acids	- Development of effective methods to reduce or eliminate background amplification - Simplification of EXPAR procedures
SPIA	- High specificity and high sensitivity - Effectively avoid contamination of amplified products - The amplification products can be monitored using a real-time amplification fluorescence curve or assessed through visible fluorescence observed under natural daylight without the need for specialized equipment	- High cost for obtaining the required enzymes - Complicated experimental procedure could lead to a longer total assay time	- Simplification of SPIA procedures - Design of
HDA	- Good sensitivity - Suitable for amplifying and detecting larger nucleic acids - Ability for analyzing long DNA targets - Supports for multiplexing - Simple primer design, easy operation	- Non-suitability for analyzing samples with less than 100 copies - Involves multiple enzymatic steps and temperature cycling - Detection is less sensitive than PCR-based methods - Commercial HDA kits are limited	- Design of affordable reagents that increase the HDA’s multiplexing capability
CPA	- High sensitivity - Easy operation - Low equipment requirements - Does not require a DNA denaturation step - Has great potential for on-site, field and *in-situ* assay applications	- Requires 5 primers - Complexity in primer design - Complexity of reaction components - Difficulties in visualization the results and the complexity of result analysis	- Integration with a simple readout method, such as lateral flow assay - Integration with other technologies, such as biosensors, to simplify the PCA procedures

IMS, immunomagnetic separation; CE, capillary electrophoresis; MCE, microchip electrophoresis, PCR, polymerase chain reaction; CRISPR, clustered regularly interspaced short palindromic repeats; ELISA, enzyme-linked immunosorbent assay; ELGA, enzyme-linked gel assay; ECL, electrochemiluminescent.

To date, numerous isothermal amplification methods have emerged as viable alternatives to the PCR-based strategy in recent years. Thus far, LAMP, RPA/RAA, RCA, and SRCA are among other isothermal amplification that have been used to detect numerous pathogens in various types of food samples. These technologies offer sensitive detection, rapid amplification, and support for multiplex amplification. In terms of specificity and sensitivity, the detection outcomes yielded by these isothermal methods are often comparable, and in certain instances, even surpass the results achieved through PCR-based approaches. Regarding the LAMP method, researchers have observed that it displays substantial resistance to reagent inhibition and is well-suited for complex samples. However, the commercialization of LAMP devices encounters challenges, including the preservation of LAMP reagent and primer stability, the resolution of contamination concerns, the management of aerosol pollution during LAMP reactions, and the mitigation of substantial noise background signals in scenarios involving multiple target sites (Du et al., 2022, Jia et al., 2023, Jiang et al., 2020, Li et al., 2021, Priya et al., 2020, Wang et al., 2020a, Xie et al., 2022a, Xiong et al., 2020, Zendrini et al., 2021, Zhang et al., 2021, Zhang et al., 2022). To address the challenge of noise background signals, Ding et al. (2019) proposed the implementation of the “Dual-Priming Isothermal Amplification (DAMP)” approach. The authors demonstrated that the employment of the DAMP approach not only reduces noise backgrounds, but also augments detection capabilities, potentially even surpassing the performance of traditional LAMP and qPCR methodologies.

Regarding the RPA/RAA assay, the manual operations and cumbersome peripheral equipment required during nucleic acid extraction, amplification, and detection present challenges that limit the widespread field-testing applications of this technology. Integrating RPA/RAA with other systems, such as microfluidics (Qi et al., 2023, Wu et al., 2022), could potentially address these challenges and result in more rapid, stable, and easy-to-operate on-site detection tools that require minimal labor, time, and energy consumption. Furthermore, the present expenses associated with utilizing RPA technology are high, rendering it inaccessible to some due to the limited availability of RPA kits sold by only two companies (Tan et al., 2022). Wang et al. (2020c) reported that the cost of using RPA was estimated to be almost five-fold higher than qPCR. As a result, RPA technology is mainly used for scientific research and is not an open technology. To enhance access to this technology and enable its widespread application in the food safety area, future studies should focus on developing the next generation of RPA technology with improved specificity by understanding its chemistry and kinetics, rather than solely relying on companies to provide RPA kits.

RCA technology shows great promise as a suitable candidate for on-site testing due to its convenient and easily accessible amplification requirements (Guo et al., 2022a, Liu et al., 2020, Liu et al., 2021, Prasad et al., 2023, Xu et al., 2021, Yuan et al., 2022, Zhan et al., 2020). However, certain drawbacks need to be addressed in the development of the RCA method, such as the background interference during signal detection, and nonspecific binding caused by the large molecular weight of RCA products, as well as the influence of complex food matrices (Guo et al., 2022a, Liu et al., 2020, Liu et al., 2021, Prasad et al., 2023, Xu et al., 2021, Yuan et al., 2022, Zhan et al., 2020). Moreover, the design and testing of padlock probes within the RCA assay are intricate and time-consuming. Interestingly, SRCA does not need padlock probes and the whole operation is relatively simpler than RCA, although these methods share similar mechanisms. Thus far, however, the availability of commercial kits tailored specifically for SRCA remains limited. Other isothermal amplification methods, including NASBA, SDA, EXPAR, SPIA, HDA, and CPA have shown promise as viable alternatives to the traditional cultured-based methods and PCR-based techniques. However, these methods have not been widely applied in detecting pathogens in food samples in the past five years. Future studies are thus suggested to explore the application of these method in detecting more pathogens in various types of food samples.

Studies have indicated that isothermal amplification methods have achieved high sensitivity and specificity in detecting foodborne pathogens within food samples. Moreover, several studies have demonstrated that certain isothermal amplification techniques can even surpass the performance of PCR-based methods. Isothermal methods, in contrast to PCR-based approaches, tend to be cost-effective, rapid, and possess the capability to seamlessly integrate with other advanced technologies like biosensors, making them viable tools for on-site or field testing. However, both thermo-cycle and isothermal amplification methods have common challenges: the interference of food matrices and false-positive results, among others. Thus far, little is known about how to eliminate interference from the food matrix. Enrichment of samples may reduce the effect of the food matrix interference but this approach will increase the duration of the analysis. False-positive results could be attributed to the amplification of DNA from dead cells, among others. Thus, researchers have attempted to develop amplification methods that are capable of differentiating between viable and non-viable microbial cells, such as amplification combined with propidium monoazide (PMA) treatment, RNA amplification, or using bacteriophage (Foddai & Grant, 2020), among others. Note that most studies involving PMA treatment were PCR-based methods (Tables S1-2), and scant information exists concerning its integration with isothermal amplification techniques. Previous studies have pointed out that employing PMA to differentiate between DNA originating from live and death cells encounters several challenges, including the influence of food matrices, heat treatment, and the concentration of bacterial cells (Lv et al., 2020, Petersen et al., 2021, Zhang et al., 2023). Therefore, the design and optimization of the treatment conditions for using PMA coupled with a certain amplification method are necessary. In addition to efforts on enabling the detection of the viable pathogen in food samples, identification and optimization of other potential viability dyes are also suggested. Previous studies have indicated that DyeTox13 and thiazole orange monoazide (TOMO) potentially allow for the suppression of DNA signals from dead cells (Chen et al., 2022, Wang et al., 2022a). Thus far, however, very little information is available about the effectiveness of these new dyes to inhibit the DNA signals from dead cells present in food samples. Moreover, viable bacteria can also be detected through RNA amplification, albeit limited to specific methods such as reverse-transcription PCR, reverse-transcription LAMP, NASBA, SMART, SPIA, RPA/RAA, and RCA. For bacteriophage treatment, it may be challenged by the complexities in implementation, interactions between bacteriophages and hosts, and the potential development of pathogen resistance (Garrido-Maestu et al., 2019, Huang et al., 2023a).

Furthermore, the lack of uniform standard specifications for analyzing the amplicon amplified by isothermal amplification is another issue that should be addressed, especially in visual detection. Perhaps, as suggested by Wang et al. (2023), employing artificial intelligence (AI) for assessing the image difference may overcome this problem. Although combining NAA methods with AI has not been tested in the food safety area, this approach has been tested in other fields (Miao et al., 2023, Xie et al., 2022b). Finally, guidelines for validating NAA-based methods for detecting microbial pathogens in foods should be established. An example of such guidelines has been provided by the US FDA to ensure that a given analytical method meets the highest possible analytical standards for its intended purpose in the US (US FDA, 2019).

### 3.2 Future outlook of NAA-based methods

Due to the time-consuming nature of the culture-based method, its resource-intensive requirements, susceptibility to contamination risks, and the need for skilled personnel to achieve enhanced outcomes, there has been a gradual shift towards the adoption of immunological-based, biosensor-based, and molecular-based methods for detecting pathogens in food samples (Kabiraz et al., 2023a). However, the immunological techniques often suffer from limitations related to antibody cross-reactions leading to false-positive results, and subsequently, challenges of low sensitivity and limited specificity arise (Kabiraz et al., 2023a). In contrast, biosensors offer advantages over traditional approaches by delivering rapid results, affordability, ease of execution, and reduced labor demand (Kabiraz et al., 2023a, Saravanan et al., 2021). Nonetheless, the reliability of biosensor results can be a concern, potentially necessitating the development of food-specific sensors or tailored analytical tools and sampling methodologies (Campàs et al., 2023, Huang et al., 2023b, Xu et al., 2021, Yin et al., 2022). Conversely, molecular methods, such as NAA-based methods, are more sensitivity and reliable compared to alternative detection techniques. To meet the demand for pathogen detection techniques that are affordable, sensitive and specific, user-friendly, rapid and robust, equipment-free, and deliverable to end users (Campbell et al., 2021), future developments in this field should focus on simplifying, integrating, and miniaturizing NAA tools to enable on-site detection platforms for timely responses. Simplification is crucial to reduce the time required for sample pre-treatment/preparation, amplification, and readout detection. Integration with other essential technologies, such as biosensors, CRISPR/Cas-based technology, microfluidic chips, and nanotechnology, may provide ultrafast NAA with higher sensitivity, rapidity, and specificity (Gao et al., 2022, Huang et al., 2023b, Kabiraz et al., 2023a, Qi et al., 2023, Saravanan et al., 2021, Wu et al., 2022, Xu et al., 2021, Yin et al., 2022, Yin et al., 2019). Finally, miniaturizing user-friendly, inexpensive, intelligent, and portable NAA tools may facilitate the widespread use of this technology for routine monitoring of microbial hazards in foods worldwide.

## 4 Promoting the widespread implementation of naa-based methods

**Fig. 4:**
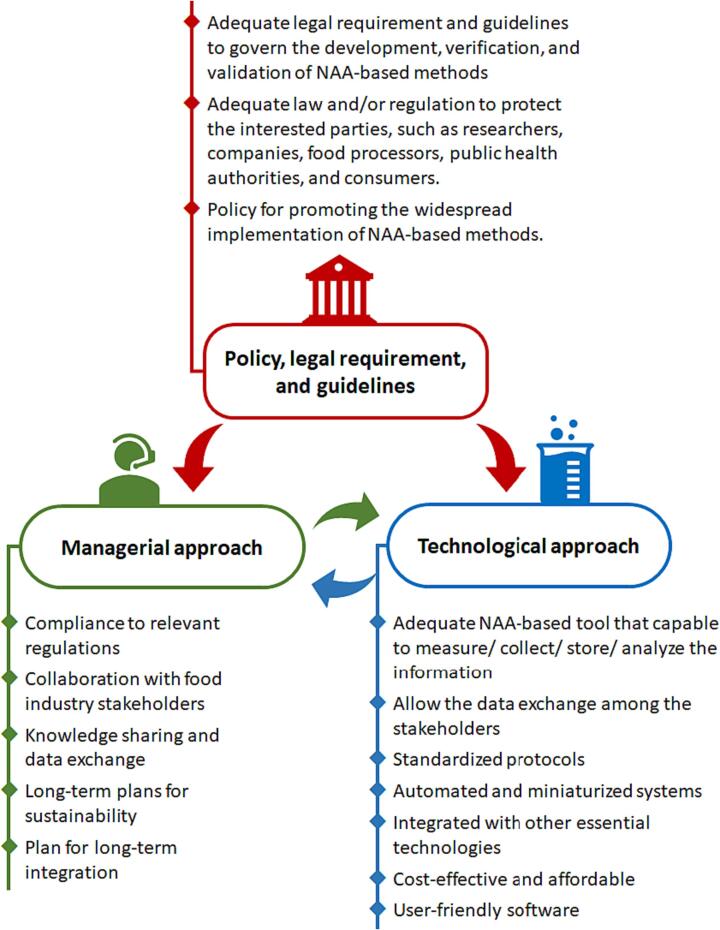
Interrelated concepts for promoting the widespread implementation of NAA-based methods in food safety areas.

To further promote the widespread implementation of NAA-based methods in supporting food safety management, it is crucial to foster collaboration and synergy among various stakeholders, an objective which can be accomplished through various approaches, including managerial and technological strategies, as well as the development of policies, legal requirements, and guidelines (Fig. 4). Government policies, regulations, or guidelines should provide more support for the widespread adoption of NAA-based methods. While some NAA-based methods have been approved in the regulations and guidelines of developed countries such as the United States and European countries (EFSA, 2023, FDA, 2023), their applications and recommended methods are often limited to popular amplification methods, such as conventional PCR or quantitative PCR. In the US, the guidelines for using PCR-based methods for detecting and/or identifying pathogens in food samples, such as *C. botulinum*, *Y. enterocolitica*, *Salmonella*, and diarrheagenic *E. coli* are available online under the “Bacteriological Analytical Manual (BAM)” on the US FDA website (Andrews et al., 2023, Feng et al., 2020, Solomon and Lilly, 2017, Weagant and Feng, 2021). Within the European Union countries, guidelines governing the utilization of PCR-based methods for pathogen detection in food samples are outlined in the “Manual for reporting on zoonoses and zoonotic agents, within the framework of Directive 2003/99/EC, and on some other pathogenic microbiological agents for information derived from the year 2021” (Amore et al., 2022). To achieve the widespread application of NAA-based methods, developing regulations or guidelines that govern the verification and validation of other amplification methods is therefore necessary. An adequate law or regulation is also necessary to protect the interested parties, such as researchers and scientist, industry and technology companies, food safety organizations, food producers and processors, as well as consumer and public health authorities. From the managerial perspective, promoting the widespread implementation of NAA-based methods involves a range of strategies and actions including fostering collaborations, seeking regulatory support, collaborating with food industry stakeholders, facilitating knowledge sharing, and developing long-term sustainability plans. By effectively implementing these measures, organizations such as government agencies responsible for food safety regulations and enforcement, public health organizations, research institutions, food testing laboratories, food manufacturers, and industry associations, can drive the adoption of NAA-based methods, leading to improved food safety practices and enhanced public health outcomes.

## 5 Conclusions

Over the past five years, significant research has been conducted to improve the sensitive, specific, and efficient detection of foodborne pathogens in food samples using thermo-cycle and isothermal NAA-based methods. However, most of the recent studies paid more attention to particular pathogens, such as *Salmonella*, *Listeria*, *E. coli*, and *V. parahaemolyticus*. Future studies are suggested to focus on detecting norovirus or other pathogens in various food samples. Additionally, future research should also evaluate the effectiveness of these methods on naturally contaminated foods, as most recent studies only focused on pathogens spiked to food samples.

In conclusion, NAA-based methods show promise for the rapid and automated detection of foodborne pathogens, albeit further improvements are necessary. The limitations of current amplification methods include false positive results. When it comes to PCR-based methods, it is worth noting that they may take longer to produce results, require specialized knowledge, and may not be practical for on-site testing in the current state. Developing thermo-cycle amplification methods with the concept of on-site testing could help to ensure a timely response. Remarkably, isothermal amplification methods have been demonstrated as potential alternatives to PCR-based methods that can offer fast, specific, and sensitive detection of pathogens in food samples.

Although NAA-based methods for detecting foodborne pathogens have shown significant improvement over the years, their adoption in the food industry and government inspection laboratories, particularly in developing countries, remains limited. This is primarily attributed to the reliance on manual procedures and complex peripheral equipment for sample pre-treatment, preparation, amplification, and detection. To address this challenge, integration with other essential technologies is essential to streamline processes such as cell lysis, DNA extraction, amplification, and detection. This integration would create more rapid, stable, and easy-to-operate on-site detection tools, requiring minimal cost, labor, time, and energy consumption. Finally, promoting the widespread implementation of NAA-based methods necessitates collaboration and synergy among various stakeholders. Through collective efforts, the practicality and accessibility of NAA-based methods can be enhanced, leading to improved food safety practices and public health outcomes.

## Declaration of Competing Interest

The authors declare that they have no known competing financial interests or personal relationships that could have appeared to influence the work reported in this paper.

## Data Availability

Data will be made available on request.
