# Supplementary material for: Rapid detection methods for foodborne pathogens based on nucleic acid amplification: Recent advances, remaining challenges, and possible opportunities
Source: Food Chem (Oxf). 2023 Sep 18;7:100183. doi: 10.1016/j.fochms.2023.100183 (PMC10520789; doi:10.1016/j.fochms.2023.100183)
Supplement: Supplementary data 1 [file mmc1.docx]

SUPPLEMENTARY INFORMATION

**Rapid detection methods for foodborne pathogens based on nucleic acid amplification: Recent advances, remaining challenges, and possible opportunities**

Nodali Ndraha ^a^, Hung-Yun Lin ^b^, Chen-Yow Wang ^a^, Hsin-I Hsiao ^c^, Han-Jia Lin ^a,b, *^

^a^ Department of Bioscience and Biotechnology, National Taiwan Ocean University, Keelung 202301, Taiwan

^b^ Center of Excellence for the Oceans, National Taiwan Ocean University, Keelung 202301, Taiwan

^c^ Department of Food Science, National Taiwan Ocean University, Keelung, 202301 Taiwan

^*^ Corresponding author: Han-Jia Lin, Department of Bioscience and Biotechnology, National Taiwan Ocean University, Keelung 202301, Taiwan. Email: [hanjia@mail.ntou.edu.tw](mailto:hanjia@mail.ntou.edu.tw)

The following supporting information is available for this article:

**Table S1**. Examples of recent studies on the development and application of conventional PCR for detecting foodborne pathogens food products.

**Table S2**. Examples of recent studies on the development and application of qPCR for detecting foodborne pathogens food products.

**Table S3**. Examples of recent studies on the development and application of loop-mediated isothermal amplification (LAMP) for detecting foodborne pathogens in food products.

**Table S4**. Examples of recent studies on the development and application of recombinase polymerase amplification (RPA) for detecting foodborne pathogens in food products.

**Table S5**. Examples of recent studies on the development and application of recombinase-aided amplification (RAA) for detecting foodborne pathogens in food products.

**Table S6**. Examples of recent studies on the development and application of rolling circle amplification (RCA) for detecting foodborne pathogens in food products.

**Table S7**. Examples of recent studies on the development and application of rolling circle amplification (SRCA) for detecting foodborne pathogens in food products.

**Table S8**. Examples of recent studies on the development and application of other types of isothermal amplification methods for detecting foodborne pathogens in food products.

Abbreviations:

**AME**, aptamer magnetic enrichment

**AuNP**, gold nanoparticle

**CE**, capillary electrophoresis;

**CRISPR**, clustered regularly interspaced short palindromic repeats

**EuNP**, europium nanoparticles

**EXPAR**, exponential amplification reaction

**HDA**, helicase-dependent amplification

**IMS**, immunomagnetic separation

**LAMP**, loop-mediated isothermal amplification

**LFA**, lateral flow assay

**LOD**, limit of detection

**MCE-LIF**, microchip electrophoresis/light-emitting diode-induced fluorescence

**MDA**, multiple displacement amplification

**MFP**, multiple fluorescent probe-based

**MNPs**, magnetic nanoparticles

**NASBA**, nucleic acid sequence-based amplification

**NR**, not reported

**PCR**, polymerase chain reaction

**PIF**, powdered infant formula

**PMA**, propidium monoazide

**qPCR**, real-time PCR

**RAA**, recombinase-aided amplification

**RCA**, rolling circle amplification

**RPA**, recombinase polymerase amplification

**SDA**, strand displacement amplification

**SDS**, sodium dodecyl sulfate

**SMART**, signal-mediated amplification of RNA technology

**SPIA**, single primer isothermal amplification

**SPRI**, solid phase reversible immobilization

**SRCA**, saltatory rolling circle amplification

**TAT**, total assay time

**TC-RCA**, target-cyclization rolling circle amplification

Table S1. Examples of recent studies on the development and application of conventional PCR for detecting foodborne pathogens food products.

| **Targeted pathogens** | **Samples** | **Enrichment** | **Reaction type** | **Combined techniques** | **LOD** | **TAT** | **References** |
| --- | --- | --- | --- | --- | --- | --- | --- |
| *V. parahaemolyticus* | Shrimp | No | Simplex | CRISPR/Cas12a | 10^2^ cfu/mL | NR | (Zhang et al., 2020) |
| *V. parahaemolyticus* | Codfish | 2 h | Simplex | PMA, LFA | 50 cfu/g | 2−4 h | (Zeng et al., 2020) |
| *E. coli* O157:H7 and *Salmonella* Typhimurium | Cabbage | No | Duplex | PMA, LFA | Ec: 10^4^ cfu/mL; ST: 10^3^ cfu/mL | 100 min | (Kim & Oh, 2021b) |
|  |  | No | Duplex | PMA, filtration, LFA | Ec: 10^3^ cfu/mL; ST: 10^2^ cfu/mL |  |  |
|  |  | No | Duplex | PMA, filtration, DNA concentration, LFA | Ec: 10^2^ cfu/mL; ST: 10 cfu/mL |  |  |
| *V. cholerae* | Shrimp | No | Simplex | Agarose gel | 10^3^ cfu/mL | NR | (Fang et al., 2021) |
| *Salmonella* spp*., Shigella* spp.*, E. coli,* norovirus | Lettuce, coriander, strawberry, and blackberry | Bacteria: 24 h, norovirus: No | Multiplex | Agarose gel | Sal, Shi, Ec: 1−10 cfu/mL; noV: 1−100 pfu/mL | ~24 h | (Hernández et al., 2022) |
| *Salmonella* Pullorum and *Salmonella* Enteritidis | Egg | No | Multiplex | Agarose gel | 10^5^ −10^6^cfu/mL | NR | (Liu et al., 2022b) |
|  |  | 2 h | Multiplex | Agarose gel | 10^4^ cfu/mL |  |  |
|  |  | 6 h | Multiplex | Agarose gel | 10 cfu/mL |  |  |
|  |  | 10−12 h | Multiplex | Agarose gel | 1 cfu/mL |  |  |
| *L. monocytogenes CC87* and *L. monocytogenes CC88* | Milk | 9 h | Multiplex | Agarose gel | Lm *CC87*: 1.9 × 10^4^ cfu/mL; Lm CC88*:* 1.7 × 10^4^ cfu/mL | NR | (Sun et al., 2022) |
| *S. aureus, E. coli* O157:H7, *Salmonella* Typhimurium, *L. monocytogenes* | Milk | No | Multiplex | CE | 10 cfu/mL | NR | (He et al., 2022) |
| *V. parahaemolyticus* | Oyster | 6−24 h | Duplex | Electrochemical biosensor | 1 cfu/mL | NR | (Campàs et al., 2023) |
| *V. parahaemolyticus, V. alginolyticus,* and *V. cholerae* | Shrimp | 10 h | Multiplex | PMA, agarose gel | 1.0 × 10^2^ cfu/g | NR | (Hu et al., 2023) |
| *V. parahaemolyticus* | Shrimp and clam | No | Simplex | Agarose gel | 7.4 × 10^4^ cfu/g | NR | (Li et al., 2023) |
|  | Goldfish | No | Simplex | Agarose gel | 7.4 × 10^5^ cfu/g |  |  |
|  | Shrimp | 2 h | Simplex | Agarose gel | 7.4 × 10^3^ cfu/g | NR |  |
|  | Shrimp | 4 h | Simplex | Agarose gel | 7.4 cfu/g | NR |  |
| *L. monocytogenes* | Milk | No | Simplex | Agarose gel | 4.4 × 10^4^ cfu/mL | NR | (Prasad et al., 2023) |
|  | Milk | 3−6 h | Simplex | Agarose gel | 4.4 × 10^3^ cfu/mL |  |  |
|  | Kalakhand | No | Simplex | Agarose gel | 9.4 × 10^2^ cfu/mL |  |  |
|  | Kalakhand | 3−6 h | Simplex | Agarose gel | 9.4 cfu/mL |  |  |

Table S2. Examples of recent studies on the development and application of qPCR for detecting foodborne pathogens food products.

| **Targeted pathogens** | **Samples** | **Enrichment** | **Reaction type** | **Combined techniques** | **LOD** | **TAT** | **References** |
| --- | --- | --- | --- | --- | --- | --- | --- |
| *Salmonella* Enteritidis | Chicken meat | 3 h | Simplex | Bacteriophage, fluorescence | 8 cfu/25 g | 10 h | (Garrido-Maestu et al., 2019) |
| *E. coli* O157:H7, *Salmonella* Typhimurium and *L. monocytogenes* | Lettuce and beef | No | Simplex | Fluorescence | Ec: 10^3^ cfu/mL; Lm, ST: 10^2^ cfu/mL | NR | (Kim et al., 2020) |
|  |  | No | Simplex | DNA concentration, fluorescence | Ec, Lm: 10^2^ cfu/mL; ST: 10 cfu/mL for *S.* Typhimurium |  |  |
|  |  | No | Simplex | Filtration, DNA concentration, fluorescence | Ec, Lm: 10 cfu/mL; ST: 1 cfu/mL |  |  |
| *E. coli O157:H7, Salmonella* Typhimurium | Lettuce and cabbage | No | Simplex | Filtration, and DNA concentration, fluorescence | 1 cfu/25 g | 2 h | (Kim & Oh, 2020) |
| *E. coli* O157:H7 | Milk, ground beef, and cabbage | 1 h | Simplex | IMS, fluorescence | 10−10^2^ cfu/mL | 3 h | (Park et al., 2020) |
| *L. monocytogenes, C. sakazakii, S. aureus,* and *Salmonella* spp*.* | Milk | No | Multiplex | SDS, PMA, fluorescence | 10^2^ cfu/mL | 7 h | (Qin et al., 2020) |
| *Salmonella* Typhimurium*, S. aureus,* and *L. monocytogenes* | Milk | No | Multiplex | IMS, SDS, PMA, fluorescence | 10 cfu/mL | 9 h | (Shi et al., 2021) |
| *E. coli O157:H7* and *S. aureus* | Milk | 4 h | Simplex | Fluorescence | 10^2^ cfu/mL | NR | (Wang et al., 2020b) |
| *Salmonella* Typhimurium*, S. aureus,* and *L. monocytogenes* | Milk | No | Multiplex | Fluorescence | 10^2^ cfu/mL | 9 h | (Shi et al., 2021) |
| *S. aureus* | Rice cereal, PIF | No | Duplex | Fluorescence | 10 cfu/mL | 2 h | (Xie & Liu, 2021) |
| *Campylobacter* and *Salmonella* | Chicken meat | No | Simplex | Fluorescence | Cp: 10^3^ cfu/mL Sal: 10 cfu/mL | NR | (Zendrini et al., 2021) |
| *E. coli* O157:H7 | Cabbage | 2 h | Simplex | Fluorescence | ≤ 7 cfu/25 g | 4 h | (Kim & Oh, 2021a) |
| *S. aureus* | Milk | No | Simplex | Fluorescence | Sau: 1.5 × 10^2^ cfu/mL | NR | (Kim et al., 2021) |
| *S. capitis* | Milk | No | Simplex | Fluorescence | 2.6 × 10^2^ cfu/mL |  | (Kim et al., 2021) |
| *S. caprae* | Milk | No | Simplex | Fluorescence | 1.4 × 10^2^ cfu/mL |  | (Kim et al., 2021) |
| *S. epidermidis* | Milk | No | Simplex | Fluorescence | 1.2 × 10^2^ cfu/mL |  | (Kim et al., 2021) |
| *L. monocytogenes, Salmonella* spp*.,* and *E. coli* O26:H11 | Pork, beef, chicken, mung bean sprout | 18 h | Multiplex | Fluorescence | 1 cfu/25 g | ~20 h | (Bundidamorn et al., 2021) |
| *E. coli* O157:H7 and *Salmonella* Typhimurium | Fresh-cut apples | No | Simplex | IMS, filtration, fluorescence | Ec: 27 cfu/mL; ST: 1.8 × 10^2^ cfu/mL | NR | (Lee et al., 2022b) |
|  |  |  |  |  |  |  |  |
| *E. coli* O157:H7 | Ground turkey | No | Simplex | IMS, fluorescence | 10 cfu/mL | NR | (Dhital & Mustapha, 2023) |
| *E. coli* O157:H7 | Chicken breast, green bell pepper, and packaged leafy greens | 4 h | Simplex | IMS, fluorescence | 10 cfu/mL |  |  |
|  | Ground beef, beef trim, ground chicken, and tomato | 6 h | Simplex | IMS, fluorescence | 10 cfu/mL |  |  |
|  | Romaine lettuce | 8 h | Simplex | IMS, fluorescence | 10 cfu/mL |  |  |
|  | Chicken breast, packaged leafy greens, and romaine lettuce | No | Simplex | SPRI, fluorescence | 10 cfu/mL |  |  |
|  | Ground beef, beef trim, ground turkey, ground chicken, green bell pepper, and tomato | 4 h | Simplex | SPRI, fluorescence | 10 cfu/mL |  |  |
| *E. coli* O157:H7 | Spinach | 8 h | Simplex | SPRI, fluorescence | 10 cfu/mL |  |  |
| *S. enterica* and *S. aureus* | Milk and lettuce | No | Duplex | Dual-phage amplification, fluorescence | 10 cfu/mL | 4 h | (Huang et al., 2023) |

Table S3. Examples of recent studies on the development and application of loop-mediated isothermal amplification (LAMP) for detecting foodborne pathogens in food products.

| **Targeted pathogens** | **Samples** | **Enrichment** | **Reaction type** | **Combined techniques** | **LOD** | **TAT** | **References** |
| --- | --- | --- | --- | --- | --- | --- | --- |
| *Salmonella* Typhimurium | Chevon | No | Simplex | Fluorescence | 8.5 cfu/g | NR | (Priya et al., 2020) |
|  |  | 6 | Simplex | Fluorescence | 2.5 cfu/g |  |  |
|  |  | 12 | Simplex | Fluorescence | 1.5 cfu/g |  |  |
| *Salmonella* spp., *Cronobacter* spp., and *S*. *aureus* | PIF | No | Multiplex | LFA | Sal: 4.2 cfu/g; Cr: 2.6 cfu/g; Stap: 3.4 cfu/g | < 1 h | (Jiang et al., 2020) |
| *S. aureus* | Fish | No | Simplex | Colorimetric | 3.2 × 10^4^ cfu/g |  | (Xiong et al., 2020) |
| *Salmonella* | Chicken meat supernatants | No | Simplex | PMA, MNPs, turbidity | 14 cfu/mL | 1.5 h | (Wang et al., 2020a) |
| *Campylobacter* | Chicken meat | No | Simplex | Colorimetric | 10^3^ cfu/mL | NR | (Zendrini et al., 2021) |
| *Salmonella* | Chicken meat | 4−6 h | Simplex | Colorimetric | 10 cfu/mL | NR | (Zendrini et al., 2021) |
| *Salmonella* spp*.* | Milk | 12 h | Simplex | Microchip | 12 cfu/mL | NR | (Zhang et al., 2021) |
| *S. enterica* and *S. aureus* | Pork | No | Simplex | Corona discharge, colorimetric | Sal: 1 cfu/mL; Stap: 10^3^ cfu/mL | NR | (Li et al., 2021) |
| *V. parahaemolyticus, S. aureus,* and *Salmonella* spp*.* | Shrimp, clam, and crab | 18 h | Multiplex | MFP, fluorescence | 5 cfu/25 g | 25 min | (Du et al., 2022) |
| *Salmonella* Typhimurium | Chicken meat | No | Simplex | Fluorescence | 55 cfu/mL | NR | (Jia et al., 2023) |
|  |  | No | Simplex | AME, RNase H2, fluorescence | 5.5 cfu/mL | NR |  |
| *S. aureus, Salmonella* Typhimurium*, E. coli* | Milk | No | Multiplex | DMF, fluorescence | 10^3^ cfu/ml | 15 min | (Xie et al., 2022) |
| *V. parahaemolyticus* | Oyster | No | Simplex | TC-RCA, fluorescence | 22 cfu/g | NR | (Zhang et al., 2022) |

Table S4. Examples of recent studies on the development and application of recombinase polymerase amplification (RPA) for detecting foodborne pathogens in food products.

| **Targeted pathogens** | **Samples** | **Enrichment** | **Reaction type** | **Combined techniques** | **LOD** | **TAT** | **References** |
| --- | --- | --- | --- | --- | --- | --- | --- |
| *S. aureus, V. parahaemolyticus,* and *Salmonella* Enteritidis | Sleeve fish | No | Multiplex | LFA | Stap: 41 cfu/mL; Vp: 79 cfu/mL; SE: 26 cfu/mL | NR | (Ma et al., 2020) |
|  | Shrimp | No | Multiplex | LFA | Stap: 42 cfu/mL; Vp: 80 cfu/mL; SE: 26 cfu/mL | NR |  |
|  | Cod | No | Multiplex | LFA | Stap: 42 cfu/mL; Vp: 78 cfu/mL; SE: 25 cfu/mL | NR |  |
| *E. coli* and *S. aureus* | Milk | 4h | Simplex | CRISPR/Cas12a | 1 cfu/mL | < 5 h | (Wang et al., 2020b) |
| *Salmonella* Typhimurium | Milk |  | Simplex | IMS, CRISPR/Cas12a | 1 cfu/mL | NR | (Cai et al., 2021) |
| *L. monocytogenes, V. parahaemolyticus* and *E. coli O157:H7* | Beef, milk, chicken breast, shrimp | No | Multiplex | Colloidal gold-based LFA | Lm: 90 cfu/mL; Vp, Ec: 70 cfu/mL |  | (Chen et al., 2021) |
|  |  |  | Multiplex | EuNP-based LFA | Lm: 9 cfu/mL; Vp: 7 cfu/mL; Ec: 4 cfu/mL | NR |  |
| *L. monocytogenes* | Milk | No | Simplex | CRISPR/Cas12a | 10 cfu/mL | NR | (Tian et al., 2021) |
| *V. cholerae and V. vulnificus* | Shrimp | 4 h | Duplex | LFA | 1 cfu/10 g | NR | (Wang et al., 2021) |
| *V. parahaemolyticus*, *S. aureus*, *S. enterica* *E. coli O157:H7*, and *Listeria monocytogenes* | Chicken, pork, beef, milk, shrimp, and fish | No | Multiplex | AuNP-based LFA | Vp: 24 cfu/mL; Stap: 71 cfu/mL; SE: 45 cfu/mL; Ec: 51 cfu/mL; Lm: 27 cfu/mL | NR | (Jin et al., 2022) |
| *Salmonella* spp. | Chicken and egg | No | Simplex | CRISPR/Cas12a | 10^3^ cfu/mL | < 4 h | (Liu et al., 2022a) |
|  | Chicken | 3 h | Simplex | CRISPR/Cas12a | 10^2^ cfu/mL |  |  |
|  | Egg | 3 h | Simplex | CRISPR/Cas12a | 10 cfu/mL |  |  |
| *E. coli O157:H7* | Ground chicken | 10 min | Simplex | LFA | 10 cfu/mL | NR | (Petrucci et al., 2022) |
| *Y. enterocolitica* | Pork | No | Simplex | CRISPR/Cas12a | 1.7 cfu/mL | NR | (Xiao et al., 2022) |

Table S5. Examples of recent studies on the development and application of recombinase-aided amplification (RAA) for detecting foodborne pathogens in food products.

| **Targeted pathogens** | **Samples** | **Enrichment** | **Reaction type** | **Combined techniques** | **LOD** | **TAT** | **References** |
| --- | --- | --- | --- | --- | --- | --- | --- |
| *S. aureus* | Milk | 3 h | Simplex | PMA, fluorescence | 10^2^ cfu/mL | NR | (Xie et al., 2021) |
|  |  | 6 h | Simplex | Fluorescence | 10 cfu/mL |  |  |
| *E. coli* O157:H7 | Milk | No | Simplex | Fluorescence | 5.4 × 10 cfu/mL | 20 min | (Mu et al., 2021) |
| *E. coli* O157:H7 | Lettuce |  | Simplex | Fluorescence | 7.0 × 10 cfu/mL |  |  |
| *V. cholerae* | Shrimp | No | Simplex | LFA | 46 cfu/mL | 50 min | (Fang et al., 2021) |
| *Salmonella* Enteritidis | Milk | 3 h | Simplex | TOMA, fluorescence | 3.5 × 10^2^ cfu/mL | NR | (Feng et al., 2022) |
| *Salmonella* Enteritidis | Milk | 5 h | Simplex | TOMA, fluorescence | 3.5 cfu/mL |  |  |
| *Salmonella* spp. | Milk | No | Simplex | PMA, fluorescence | 1.8 × 10^3^ cfu/mL | 30 min | (Li et al., 2022) |
| *E. coli* O157:H7 | Milk | No | Simplex | Fluorescence | 7.9 cfu/mL | NR | (Mu et al., 2022) |
| *Salmonella T*yphimurium | Chicken meat | No | Simplex | Fluorescence | 10 cfu/mL | 1 h | (Wang et al., 2022) |
| *Salmonella T*yphimurium | Pork | No | Simplex | Fluorescence | 89 cfu/mL | 40 min | (Wu et al., 2022) |
| *C. jejuni* | Chicken meat | 24 h | Simplex | CRISPR/Cas12a | 1.2 cfu/mL | NR | (Zhi et al., 2022) |
|  |  | 48 h | Simplex | CRISPR/Cas12a | 0.12 cfu/mL |  |  |
|  |  |  |  | Fluorescence | 0.12 cfu/mL |  |  |
| *Salmonella T*yphimurium | Chicken meat | No | Simplex | PMA, fluorescence | 1.3 × 10 cfu/mL | 2 h | (Qi et al., 2023) |
| *V. parahaemolyticus* | Shrimp and clam | No | Simplex | LFA | 7.4 × 10^5^ cfu/g | NR | (Li et al., 2023) |
|  | Goldfish | No | Simplex | LFA | 7.4 × 10^4^ cfu/g | NR | (Li et al., 2023) |
|  | Shrimp | 2 h | Simplex | LFA | 7.4 × 10^3^ cfu/g | NR | (Li et al., 2023) |
|  | Shrimp | 4 h | Simplex | LFA | 7.4 cfu/g | NR | (Li et al., 2023) |

Table S6. Examples of recent studies on the development and application of rolling circle amplification (RCA) for detecting foodborne pathogens in food products.

| **Targeted pathogens** | **Samples** | **Enrichment** | **Reaction type** | **Combined techniques** | **LOD** | **TAT** | **References** |
| --- | --- | --- | --- | --- | --- | --- | --- |
| *Cronobacter* spp*.* | Milk | No | Simplex | Fluorescence | 4.5 × 10^2^ cfu/mL | NR | (Liu et al., 2020) |
| *L. monocytogenes* | Lettuce | No | Simplex | Colorimetric | 6.1 × 10^3^ cfu/mL | NR | (Zhan et al., 2020) |
| *C. sakazakii* | PIF | No | Simplex | Colorimetric | 2.4 × 10^3^ cfu/mL | NR | (Liu et al., 2021b) |
| *Cronobacter* spp*.* | PIF | No | Simplex | Fluorescence | 9.2 × 10^2^ cfu/mL | NR | (Liu et al., 2021a) |
|  | PIF | No | Simplex | Colorimetric | 8.4 × 10^3^ cfu/mL | NR |  |
|  | PIF | 5−7 h | Simplex | Fluorescence and colorimetric | 1 cfu/mL | NR |  |
| *Salmonella* Typhimurium and *S. flexneri* | Milk | No | Duplex | Fluorescence | 10 cfu/mL | NR | (Guo et al., 2022a) |
| *V. parahaemolyticus* | Oyster | No | Simplex | Fluorescence | 2.3 cfu/g | NR | (Yuan et al., 2022) |
|  | Oyster | No | Simplex | Agarose gel | 23 cfu/g | NR |  |
| *L. monocytogenes* | Milk | No | Simplex | Fluorescence | 4.4 × 10^2^ cfu/mL | NR | (Prasad et al., 2023) |
|  | Kalakhand | No | Simplex | Fluorescence | 9.4 × 10^2^ cfu/mL |  |  |
|  | Milk | 3−6 h | Simplex | Fluorescence | 4.4 cfu/mL |  |  |
|  | Kalakhand | 3−6 h | Simplex | Fluorescence | 9.4 cfu/mL |  |  |

Table S7. Examples of recent studies on the development and application of SRCA for detecting foodborne pathogens in food products.

| **Targeted pathogens** | **Samples** | **Enrichment** | **Reaction type** | **Combined techniques** | **LOD** | **TAT** | **References** |
| --- | --- | --- | --- | --- | --- | --- | --- |
| *C. sakazakii* | PIF | No | Simplex | Gel electrophoresis, precipitation | 340 cfu/mL | NR | (Zhang et al., 2019) |
| *S. aureus* | Milk | No | Simplex | Gel electrophoresis, precipitation | 560 cfu/mL | NR | (Yang et al., 2019) |
|  | Milk | No | Simplex | Fluorescence | 56 cfu/mL |  |  |
| *Cronobacter* spp. | PIF | No | Simplex | Colorimetric | 8.4 × 10^3^ cfu/mL |  | (Liu et al., 2021a) |
|  | PIF | No | Simplex | Fluorescence | 9.2 × 10^2^ cfu/mL |  |  |
| *Campylobacter jejuni* | Chicken meat | No | Simplex | Fluorescence | 10^3^ cfu/g | 120 min | (Milton et al., 2021a) |
|  | Chicken meat | 12 h | Simplex | Fluorescence | 10^2^ cfu/g | 14 h |  |
| *Salmonella* | Pork | No | Simplex | Fluorescence | 40 cfu/g | 165 min | (Milton et al., 2021b) |
| *C. perfringens* | Pork | No | Simplex | Fluorescence | 80 cfu/g | NR | (Milton et al., 2021c) |
| *Salmonella* spp. and *Shigella* spp. | Milk | No | Multiplex | Fluorescence | 10 cfu/mL | NR | (Guo et al., 2022a) |
| *S. aureus* | Pork and pork products | No | Simplex | PMA, gel electrophoresis | 660 cfu/g | NR | (Guo et al., 2022b) |
|  |  |  |  | Fluorescence | 66 cfu/g |  |  |
|  |  |  |  |  |  |  |  |
| *V. parahaemolyticus* | Oyster | No | Simplex | Real-time fluorescence | 2.3 cfu/g | 20−60 min | (Yuan et al., 2022) |
| *L. monocytogenes* | Milk | No | Simplex | Fluorescence | 440 cfu/mL | NR | (Prasad et al., 2023) |
|  | Milk | 3−6 h | Simplex | Fluorescence | 4.4 cfu/mL | NR |  |
|  | Kalakhand | No | Simplex | Fluorescence | 940 cfu/mL | NR |  |

Table S8. Examples of recent studies on the development and application of other types of isothermal amplification for detecting foodborne pathogens in food products.

| **Targeted pathogens** | **Samples** | **Amplification method** | **Enrichment** | **Reaction type** | **Combined techniques** | **LOD** | **TAT** | **References** |
| --- | --- | --- | --- | --- | --- | --- | --- | --- |
| *Salmonella* | Pork | NASBA | 12 h | Simplex | Fluorescent beacon | 9.5 × 10^3^ cfu/mL | NR | (Zhai et al., 2019) |
| *Salmonella* Paratyphi C | Pork and chicken | NASBA | 12 h | Simplex | Fluorescence | 4.9 cfu/25 g | NR | (Zhai et al., 2022) |
| *S. aureus* | Milk | SDA |  | Simplex | Fluorescence | 1 × 10^4^ cfu/mL | NR | (Cai et al., 2020) |
| *C. sakazakii* | Milk | EXPAR | No | Simplex | IMB, aptamer, colorimetric | 12 cfu/g | <2 h | (Xu et al., 2022) |
| *L. monocytogenes* | Chicken meat | SPIA | No | Simplex | Fluorescence | 1.4 cfu/g | 30−60 min | (Yang et al., 2020) |
| *V. parahaemolyticus* | Oyster | SPIA | No | Simplex | Fluorescence | 42 cfu/g |  | (Yang et al., 2021) |
| *Salmonella* | Pork | SPIA | No | Simplex | Electrochemical biosensor | 68 cfu/g | 1 h | (Yin et al., 2022) |
| Norovirus | Oyster | HDA | 3 h | Simplex | Colorimetric | 10^2^ copies/mL | 2 h | (Lee et al., 2022a) |
| *Escherichia coli* O157:H7 | Fresh salad mix | HDA | No | Simplex | Filtration, CRISPR/Cas12a, fluorescence | 10^3^ cfu/mL | NR | (Kim et al., 2023) |
| *E. coli* O157:H7 | Cantonese rice cake | CPA | No | Simplex | Gel electrophoresis | 10^3^−10^5^ cfu/mL | 1 h | (Xu et al., 2020) |

.

**References**

Bundidamorn, D., Supawasit, W., & Trevanich, S. (2021). Taqman® probe based multiplex RT-PCR for simultaneous detection of *Listeria monocytogenes*, *Salmonella* spp. and Shiga toxin-producing *Escherichia coli* in foods. *LWT*, *147*, 111696. https://doi.org/10.1016/j.lwt.2021.111696

Cai, Q., Wang, R., Qiao, Z., & Yang, W. (2021). Single-digit *Salmonella* detection with the naked eye using bio-barcode immunoassay coupled with recombinase polymerase amplification and a CRISPR-Cas12a system. *Analyst*, *146*, 5271–5279. https://doi.org/10.1039/d1an00717c

Cai, R., Yin, F., Chen, H., Tian, Y., & Zhou, N. (2020). A fluorescent aptasensor for *Staphylococcus aureus* based on strand displacement amplification and self-assembled DNA hexagonal structure. *Microchimica Acta*, *187*, 304. https://doi.org/10.1007/s00604-020-04293-9

Campàs, M., Leonardo, S., Ferré-Gode, A., Chowdhury, A. A., Toldrà, A., Andree, K. B., & Roque, A. (2023). Duplex electrochemical biosensor for the detection of the *tdh* and *trh* virulence genes of *Vibrio parahaemolyticus* in oysters. *Food Control*, 109689. https://doi.org/10.1016/j.foodcont.2023.109689

Chen, K., Ma, B., Li, J., Chen, E., Xu, Y., Yu, X., Sun, C., & Zhang, M. (2021). A rapid and sensitive europium nanoparticle-based lateral flow immunoassay combined with recombinase polymerase amplification for simultaneous detection of three food-borne pathogens. *International Journal of Environmental Research and Public Health*, *18*, 4574. https://doi.org/10.3390/ijerph18094574

Dhital, R., & Mustapha, A. (2023). DNA concentration by solid phase reversible immobilization improves its yield and purity, and detection time of *E. coli* O157:H7 in foods by high resolution melt curve qPCR. *Food Control*, *145*, 109456. https://doi.org/10.1016/j.foodcont.2022.109456

Du, J., Ma, B., Li, J., Shuai, J., Yu, X., Zhang, X., & Zhang, M. (2022). Probe-based loop-mediated isothermal amplification assay for multi-target quantitative detection of three foodborne pathogens in seafood. *Food Analytical Methods*, *15*, 3479–3489. https://doi.org/10.1007/s12161-022-02381-5

Fang, W., Cai, Y., Zhu, L., Wang, H., & Lu, Y. (2021). Rapid and highly sensitive detection of toxigenic Vibrio cholerae based on recombinase-aided amplification combining with lateral flow assay. *Food Analytical Methods*, *14*, 687–696. https://doi.org/10.1007/s12161-020-01909-x

Feng, X., Zhou, D., Gan, B., Xie, G., & Xu, H. (2022). A combination of novel nucleic acid cross-linking dye and recombinase-aided amplification for the rapid detection of viable *Salmonella* in milk. *Foods*, *11*, 2375. https://doi.org/10.3390/foods11152375

Garrido-Maestu, A., Fuciños, P., Azinheiro, S., Carvalho, C., Carvalho, J., & Prado, M. (2019). Specific detection of viable *Salmonella* Enteritidis by phage amplification combined with qPCR (PAA-qPCR) in spiked chicken meat samples. *Food Control*, *99*, 79–83. https://doi.org/10.1016/j.foodcont.2018.12.038

Guo, W., Yang, Q., Liu, J., Chen, X., Zhang, Y., & Zhang, W. (2022a). Multiple fluorescent saltatory rolling circle amplification (SRCA) for simultaneous and sensitive detection of *Salmonella* spp. and *Shigella* spp. in food. *LWT*, *168*, 113875. https://doi.org/10.1016/j.lwt.2022.113875

Guo, W., Yang, Q., Zhang, Y., Lu, X., Wu, C., Tan, J., & Zhang, W. (2022b). Rapid and visual detection of viable *Staphylococcus aureus* in pork and pork products by PMA and saltatory rolling circle amplification. *European Food Research and Technology*, *248*, 1625–1634. https://doi.org/10.1007/s00217-022-03990-4

He, S., Huang, Y., Ma, Y., Yu, H., Pang, B., Liu, X., Yin, C., Wang, X., Wei, Y., Tian, Y., Zhao, C., Xu, K., Wang, J., Lv, C., Song, X., & Jin, M. (2022). Detection of four foodborne pathogens based on magnetic separation multiplex PCR and capillary electrophoresis. *Biotechnology Journal*, *17*, 2100335. https://doi.org/10.1002/biot.202100335

Hernández, O. H., Gutiérrez-Escolano, A. L., Cancio-Lonches, C., Iturriaga, M. H., Pacheco-Aguilar, J. R., Morales-Rayas, R., & Arvizu-Medrano, S. M. (2022). Multiplex PCR method for the detection of human norovirus, Salmonella spp., Shigella spp., and shiga toxin producing Escherichia coli in blackberry, coriander, lettuce and strawberry. *Food Microbiology*, *102*, 103926. https://doi.org/10.1016/j.fm.2021.103926

Hu, Y., Wang, W., Turmidzi, F., Li, F., Fang, L., Zhou, Z., & Zhang, D. (2023). Rapid and simultaneous detection of viable *Vibrio parahaemolyticus*, *Vibrio alginolyticus*, and *Vibrio cholerae* by PMA-mPCR assay in aquatic products. *LWT*, 114663. https://doi.org/10.1016/j.lwt.2023.114663

Huang, C., Zheng, R., Ding, Y., R. Nugen, S., & Wang, X. (2023). Dual phage amplification-mediated multiplex detection strategies for the simultaneous detection of *Salmonella enterica* and *Staphylococcus aureus*. *Talanta*, *253*, 124095. https://doi.org/10.1016/j.talanta.2022.124095

Jia, K., Xiao, R., Lin, Q., Gou, H., Peng, J., Liang, Y., Shen, H., Cai, M., Liao, M., Zhang, J., & Han, Y. (2023). RNase H2 triggered visual loop-mediated isothermal amplification combining smartphone assisted all-in-one aptamer magnetic enrichment device for ultrasensitive culture-independent detection of *Salmonella* Tphimurium in chicken meat. *Sensors and Actuators B: Chemical*, *380*, 133399. https://doi.org/10.1016/j.snb.2023.133399

Jiang, Y., Chen, S., Zhao, Y., Yang, X., Fu, S., McKillip, J. L., Fox, E. M., & Man, C. (2020). Multiplex loop-mediated isothermal amplification-based lateral flow dipstick for simultaneous detection of 3 food-borne pathogens in powdered infant formula. *Journal of Dairy Science*, *103*, 4002–4012. https://doi.org/10.3168/jds.2019-17538

Jin, B., Ma, B., Li, J., Hong, Y., & Zhang, M. (2022). Simultaneous detection of five foodborne pathogens using a mini automatic nucleic acid extractor combined with recombinase polymerase amplification and lateral flow immunoassay. *Microorganisms*, *10*, 1352. https://doi.org/10.3390/microorganisms10071352

Kim, E., Yang, S.-M., Won, J.-E., Kim, D.-Y., Kim, D.-S., & Kim, H.-Y. (2021). Real-Time PCR method for the rapid detection and quantification of pathogenic *Staphylococcus* species based on novel molecular target genes. *Foods*, *10*, 2839. https://doi.org/10.3390/foods10112839

Kim, J.-H., Jung, S., & Oh, S.-W. (2020). Combination of bacteria concentration and DNA concentration for rapid detection of *E. coli* O157:H7, *L. monocytogenes*, and *S. Typhimurium* without microbial enrichment. *LWT*, *117*, 108609. https://doi.org/10.1016/j.lwt.2019.108609

Kim, J.-H., & Oh, S.-W. (2020). Rapid and sensitive detection of *E. coli* O157:H7 and *S*. Typhimurium in iceberg lettuce and cabbage using filtration, DNA concentration, and qPCR without enrichment. *Food Chemistry*, *327*, 127036. https://doi.org/10.1016/j.foodchem.2020.127036

Kim, J.-H., & Oh, S.-W. (2021a). Rapid detection for low numbers of *Escherichia coli* O157:H7 by real-time PCR in cabbage using a combination of filtration, short microbial enrichment, and DNA concentration within 4 h. *LWT*, *139*, 110520. https://doi.org/10.1016/j.lwt.2020.110520

Kim, J. H., & Oh, S. W. (2021b). A colorimetric lateral flow assay based on multiplex PCR for the rapid detection of viable *Escherichia coli* O157:H7 and *Salmonella* Typhimurium without enrichment. *LWT*, *152*, 112242. https://doi.org/10.1016/J.LWT.2021.112242

Kim, U., Lee, S.-Y., & Oh, S.-W. (2023). Thermophilic helicase-dependent amplification-based CRISPR/Cas12a system: Detection of stx2 in *Escherichia coli* O157:H7 by controlling primer dimers. *Analytica Chimica Acta*, *1239*, 340679. https://doi.org/10.1016/j.aca.2022.340679

Lee, J.-E., Kim, S.-A., Park, H.-J., Mun, H., Ha, K.-S., & Shim, W.-B. (2022a). Colorimetric detection of norovirus by helicase-dependent amplification method based on specific primers integrated with HRPzyme. *Analytical and Bioanalytical Chemistry*, *414*, 6723–6733. https://doi.org/10.1007/s00216-022-04247-5

Lee, S.-Y., Kim, J.-H., & Oh, S.-W. (2022b). Combination of filtration and immunomagnetic separation based on real-time PCR to detect foodborne pathogens in fresh-cut apple. *Journal of Microbiological Methods*, *201*, 106577. https://doi.org/10.1016/j.mimet.2022.106577

Li, D., Zhao, J., Lan, W., Zhao, Y., & Sun, X. (2023). Effect of food matrix on rapid detection of *Vibrio parahaemolyticus* in aquatic products based on *toxR* gene. *World Journal of Microbiology and Biotechnology*, *39*, 188. https://doi.org/10.1007/s11274-023-03640-1

Li, J., Zhou, D., Xie, G., Deng, M., Feng, X., & Xu, H. (2022). PMAxx combined with recombinase aided amplification technique for specific and rapid detection of *Salmonella* in milk. *Food Analytical Methods*, *15*, 1769–1777. https://doi.org/10.1007/s12161-022-02249-8

Li, M., Luan, Z., Liu, Y., Yang, C., Wang, Y., Ma, C., & Shi, C. (2021). Ultrafast bacterial cell lysis using a handheld corona treater and loop-mediated isothermal amplification for rapid detection of foodborne pathogens. *Food Control*, *128*, 108178. https://doi.org/10.1016/j.foodcont.2021.108178

Liu, J., Xie, G., Xiong, Q., Liang, T., & Xu, H. (2021a). Sensitive dual readout assays based on rolling circle amplification for fluorescent and colorimetric detection of *Cronobacter* spp. in powdered infant formula. *Food Control*, *124*, 107840. https://doi.org/10.1016/j.foodcont.2020.107840

Liu, J., Xie, G., Xiong, Q., Mu, D., & Xu, H. (2021b). A simple and sensitive aptasensor with rolling circle amplification for viable *Cronobacter sakazakii* detection in powdered infant formula. *Journal of Dairy Science*, *104*, 12365–12374. https://doi.org/10.3168/jds.2021-20898

Liu, J., Zhan, Z., Liang, T., Xie, G., Aguilar, Z. P., & Xu, H. (2020). Dual-signal amplification strategy: Universal asymmetric tailing-PCR triggered rolling circle amplification assay for fluorescent detection of *Cronobacter* spp. in milk. *Journal of Dairy Science*, *103*, 3055–3065. https://doi.org/10.3168/jds.2019-17590

Liu, L., Zhao, G., Li, X., Xu, Z., Lei, H., & Shen, X. (2022a). Development of rapid and easy detection of *Salmonella* in food matrics using RPA-CRISPR/Cas12a method. *LWT*, *162*, 113443. https://doi.org/10.1016/j.lwt.2022.113443

Liu, Z., Yu, Y., Fotina, T., Petrov, R., Klishchova, Z., Fotin, A., & Ma, J. (2022b). Multiplex PCR assay based on the *citE2* gene and intergenic sequence for the rapid detection of *Salmonella* Pullorum in chickens. *Poultry Science*, *101*, 101981. https://doi.org/10.1016/j.psj.2022.101981

Ma, B., Li, J., Chen, K., Yu, X., Sun, C., & Zhang, M. (2020). Multiplex recombinase polymerase amplification assay for the simultaneous detection of three foodborne pathogens in seafood. *Foods*, *9*, 278. https://doi.org/10.3390/foods9030278

Milton, A. A. P., Momin, K. M., Priya, G. B., Das, S., Angappan, M., Sen, A., Sinha, D. K., & Ghatak, S. (2021a). Novel saltatory rolling circle amplification assay for rapid and visual detection of *Campylobacter jejuni* in chicken meat. *LWT*, *149*, 111807. https://doi.org/10.1016/j.lwt.2021.111807

Milton, A. A. P., Momin, K. M., Priya, G. B., Ghatak, S., Das, S., Gandhale, P. N., Angappan, M., & Sen, A. (2021b). Development of novel visual detection methodology for *Salmonella* in meat using saltatory rolling circle amplification. *Journal of Applied Microbiology*, *131*, 2361–2371. https://doi.org/10.1111/jam.15099

Milton, A. A. P., Momin, K. M., Priya, G. B., Ghatak, S., Gandhale, P. N., Angappan, M., Das, S., & Sen, A. (2021c). A novel in situ methodology for visual detection of *Clostridium perfringens* in pork harnessing saltatory rolling circle amplification. *Anaerobe*, *69*, 102324. https://doi.org/10.1016/j.anaerobe.2021.102324

Mu, D., Zhou, D., Xie, G., Liu, J., Wang, Z., Xiong, Q., & Xu, H. (2022). Real-time recombinase-aided amplification with improved propidium monoazide for the rapid detection of viable *Escherichia coli* O157:H7 in milk. *Journal of Dairy Science*, *105*, 1028–1038. https://doi.org/10.3168/jds.2021-21074

Mu, D., Zhou, D., Xie, G., Liu, J., Xiong, Q., Feng, X., & Xu, H. (2021). The fluorescent probe-based recombinase-aided amplification for rapid detection of *Escherichia coli* O157:H7. *Molecular and Cellular Probes*, *60*, 101777. https://doi.org/10.1016/j.mcp.2021.101777

Park, J. Y., Lim, M.-C., Park, K., Ok, G., Chang, H.-J., Lee, N., Park, T. J., & Choi, S.-W. (2020). Detection of *E. coli* O157:H7 in food using automated immunomagnetic separation combined with real-time PCR. *Processes*, *8*, 908. https://doi.org/10.3390/pr8080908

Petrucci, S., Dikici, E., Daunert, S., & Deo, S. K. (2022). Isothermal amplification and lateral flow nucleic acid test for the detection of shiga toxin-producing bacteria for food monitoring. *Chemosensors*, *10*, 210. https://doi.org/10.3390/chemosensors10060210

Prasad, M. C. B. C. B., Milton, A. A. P. A. P., Menon, V. K. K., Ghatak, S., Srinivas, K., Momin, K. M. M., Vineesha, S. L. L., Das, S., Sen, A., Latha, C., Sunil, B., & Jolly, D. (2023). Saltatory rolling circle amplification assay for simple and visual detection of *Listeria monocytogenes* in milk and milk products. *International Dairy Journal*, *137*, 105498. https://doi.org/10.1016/j.idairyj.2022.105498

Priya, G. B., Agrawal, R. K., Prince Milton, A. A., Mishra, M., Mendiratta, S. K., Luke, A., Inbaraj, S., Singh, B. R., Kumar, D., Kumar, G. R., & Rajkhowa, S. (2020). Rapid and visual detection of *Salmonella* in meat using invasin A (*invA*) gene-based loop-mediated isothermal amplification assay. *LWT*, *126*, 109262. https://doi.org/10.1016/j.lwt.2020.109262

Qi, W., Wang, S., Wang, L., Xi, X., Wu, S., Li, Y., Liao, M., & Lin, J. (2023). A portable viable *Salmonella* detection device based on microfluidic chip and recombinase aided amplification. *Chinese Chemical Letters*, *34*, 107360. https://doi.org/10.1016/j.cclet.2022.03.083

Qin, H., Shi, X., Yu, L., Li, K., Wang, J., Chen, J., Yang, F., Xu, H., & Xu, H. (2020). Multiplex real-time PCR coupled with sodium dodecyl sulphate and propidium monoazide for the simultaneous detection of viable *Listeria monocytogenes*, *Cronobacter sakazakii*, *Staphylococcus aureus* and *Salmonella* spp. in milk. *International Dairy Journal*, *108*, 104739. https://doi.org/10.1016/j.idairyj.2020.104739

Shi, X., Yu, L., Lin, C., Li, K., Chen, J., & Qin, H. (2021). Biotin exposure–based immunomagnetic separation coupled with sodium dodecyl sulfate, propidium monoazide, and multiplex real-time PCR for rapid detection of viable Salmonella Typhimurium, Staphylococcus aureus, and Listeria monocytogenes in milk. *Journal of Dairy Science*, *104*, 6588–6597. https://doi.org/10.3168/jds.2020-19887

Sun, Q., Cheng, J., Lin, R., Li, J., Zhang, Y., Liang, X., Su, Y., Pang, R., Xue, L., Zeng, H., Gu, Q., Ding, Y., Wu, Q., Chen, M., & Zhang, J. (2022). A novel multiplex PCR method for simultaneous identification of hypervirulent *Listeria monocytogenes* clonal complex 87 and CC88 strains in China. *International Journal of Food Microbiology*, *366*, 109558. https://doi.org/10.1016/j.ijfoodmicro.2022.109558

Tian, Y., Liu, T., Liu, C., Xu, Q., Fang, S., Wu, Y., Wu, M., & Liu, Q. (2021). An ultrasensitive and contamination-free on-site nucleic acid detection platform for *Listeria monocytogenes* based on the CRISPR-Cas12a system combined with recombinase polymerase amplification. *LWT*, *152*, 112166. https://doi.org/10.1016/j.lwt.2021.112166

Wang, P., Liao, L., Ma, C., Zhang, X., Yu, J., Yi, L., Liu, X., Shen, H., Gao, S., & Lu, Q. (2021). Duplex on-site detection of *Vibrio cholerae* and *Vibrio vulnificus* by recombinase polymerase amplification and three-segment lateral flow strips. *Biosensors*, *11*, 151. https://doi.org/10.3390/bios11050151

Wang, S., Cai, G., Duan, H., Qi, W., & Lin, J. (2022). Automatic and multi-channel detection of bacteria on a slidable centrifugal disc based on FTA card nucleic acid extraction and recombinase aided amplification. *Lab on a Chip*, *22*, 80–89. https://doi.org/10.1039/D1LC00915J

Wang, S., Liu, N., Zheng, L., Cai, G., & Lin, J. (2020a). A lab-on-chip device for the sample-in-result-out detection of viable *Salmonella* using loop-mediated isothermal amplification and real-time turbidity monitoring. *Lab on a Chip*, *20*, 2296–2305. https://doi.org/10.1039/D0LC00290A

Wang, Y., Ke, Y., Liu, W., Sun, Y., & Ding, X. (2020b). A one-pot toolbox based on Cas12a/crRNA enables rapid foodborne pathogen detection at attomolar level. *ACS Sensors*, *5*, 1427–1435. https://doi.org/10.1021/acssensors.0c00320

Wu, S., Duan, H., Zhang, Y., Wang, S., Zheng, L., Cai, G., Lin, J., & Yue, X. (2022). A *Salmonella* microfluidic chip combining non-contact eddy heater and 3D fan-shaped mixer with recombinase aided amplification. *Biosensors*, *12*, 726. https://doi.org/10.3390/bios12090726

Xiao, Y., Ren, H., Hu, P., Wang, Y., Wang, H., Li, Y., Feng, K., Wang, C., Cao, Q., Guo, Y., Liu, Z., & Lu, S. (2022). Ultra-sensitive and rapid detection of pathogenic *Yersinia enterocolitica* based on the CRISPR/Cas12a nucleic acid identification platform. *Foods*, *11*, 2160. https://doi.org/10.3390/foods11142160

Xie, G., Zhou, D., Zhao, G., Feng, X., Aguilar, Z. P., & Xu, H. (2021). Recombinase aided amplification with photoreactive DNA-binding dye for rapid detection of viable *Staphylococcus aureus*. *LWT*, *135*, 110249. https://doi.org/10.1016/j.lwt.2020.110249

Xie, M., Chen, T., Xin, X., Cai, Z., Dong, C., & Lei, B. (2022). Multiplex detection of foodborne pathogens by real-time loop-mediated isothermal amplification on a digital microfluidic chip. *Food Control*, *136*, 108824. https://doi.org/10.1016/j.foodcont.2022.108824

Xie, X., & Liu, Z. (2021). Simultaneous enumeration of *Cronobacter sakazakii* and *Staphylococcus aureus* in powdered infant foods through duplex TaqMan real-time PCR. *International Dairy Journal*, *117*, 105019. https://doi.org/10.1016/j.idairyj.2021.105019

Xiong, J., Huang, B., Xu, J., & Huang, W. (2020). A closed-tube loop-mediated isothermal amplification assay for the visual detection of *Staphylococcus aureus*. *Applied Biochemistry and Biotechnology*, *191*, 201–211. https://doi.org/10.1007/s12010-020-03278-x

Xu, X., Zhu, L., Wang, X., Lan, X., Chu, H., Tian, H., & Xu, W. (2022). Sandwich capture ultrasensitive sensor based on biohybrid interface for the detection of *Cronobacter sakazakii*. *Applied Microbiology and Biotechnology*, *106*, 4287–4296. https://doi.org/10.1007/s00253-022-11978-z

Xu, Z., Luo, Y., Soteyome, T., Lin, C.-W., Xu, X., Mao, Y., Su, J., & Liu, J. (2020). Rapid detection of food-borne Escherichia coli O157:H7 with visual inspection by crossing priming amplification (CPA). *Food Analytical Methods*, *13*, 474–481. https://doi.org/10.1007/s12161-019-01651-z

Yang, Q., Guo, W., Liu, Y., Zhang, Y., Ming, R., Yuan, Y., Tan, J., & Zhang, W. (2021). Novel Single Primer Isothermal Amplification Method for the Visual Detection of Vibrio parahaemolyticus. *FOOD ANALYTICAL METHODS*, *14*, 1995–2002. https://doi.org/10.1007/s12161-021-02033-0

Yang, Q., Xu, H., Zhang, Y., Liu, Y., Lu, X., Feng, X., Tan, J., Zhang, S., & Zhang, W. (2020). Single primer isothermal amplification coupled with SYBR Green II: Real-time and rapid visual method for detection of Listeria monocytogenes in raw chicken. *LWT*, *128*, 109453. https://doi.org/10.1016/j.lwt.2020.109453

Yang, Q., Zhang, Y., Li, S., Lu, X., Yuan, Y., & Zhang, W. (2019). Saltatory rolling circle amplification for sensitive visual detection of *Staphylococcus aureus* in milk. *Journal of Dairy Science*, *102*, 9702–9710. https://doi.org/10.3168/jds.2019-16724

Yin, C., Yuan, N., Zhang, Y., Guo, W., Liu, J., Yang, Q., & Zhang, W. (2022). Electrochemical biosensor based on single primer isothermal amplification (SPIA) for sensitive detection of *Salmonella* in food. *Food Analytical Methods*, *15*, 3270–3282. https://doi.org/10.1007/s12161-022-02387-z

Yuan, N., Yang, H., Zhang, Y., Xu, H. H., Lu, X., Xu, H. H., & Zhang, W. (2022). Development of real‐time fluorescence saltatory rolling circle amplification for rapid detection of *Vibrio parahaemolyticus* in seafood. *International Journal of Food Science & Technology*, *57*, 610–618. https://doi.org/10.1111/ijfs.15285

Zendrini, A., Carta, V., Filipello, V., Ragni, L., Cosciani-Cunico, E., Arnaboldi, S., Bertasi, B., Franceschi, N., Ajmone-Marsan, P., De Medici, D., & Losio, M. N. (2021). One-day molecular detection of *Salmonella* and *Campylobacter* in chicken meat: A pilot study. *Foods*, *10*, 1132. https://doi.org/10.3390/foods10051132

Zeng, D., Chen, S., Jiang, L., Ren, J., Ling, N., Su, J., Zhao, Y., Jiang, Y., Xue, F., Tang, F., Chen, W., Li, B., Dai, J., Han, F., & Yu, X. (2020). A polymerase chain reaction based lateral flow test strip with propidium monoazide for detection of viable *Vibrio parahaemolyticus* in codfish. *Microchemical Journal*, *159*, 105418. https://doi.org/https://doi.org/10.1016/j.microc.2020.105418

Zhai, L., Liu, H., Chen, Q., Lu, Z., Zhang, C., Lv, F., & Bie, X. (2019). Development of a real-time nucleic acid sequence–based amplification assay for the rapid detection of *Salmonella* spp. from food. *Brazilian Journal of Microbiology*, *50*, 255–261. https://doi.org/10.1007/s42770-018-0002-9

Zhai, L., Liu, H., Li, J., Lu, Z., & Bie, X. (2022). A duplex real-time NASBA assay targeting a serotype-specific gene for rapid detection of viable Salmonella Paratyphi C in retail foods of animal origin. *Canadian Journal of Microbiology*, *68*, 259–268. https://doi.org/10.1139/cjm-2021-0054

Zhan, Z., Li, H., Liu, J., Xie, G., Xiao, F., Wu, X., Aguilar, Z. P., & Xu, H. (2020). A competitive enzyme linked aptasensor with rolling circle amplification (ELARCA) assay for colorimetric detection of *Listeria monocytogenes* . *Food Control*, *107*, 106806. https://doi.org/10.1016/j.foodcont.2019.106806

Zhang, B., Sun, W., Ran, L., Wang, C., Wang, J., An, R., & Liang, X. (2022). Anti-interference detection of *Vibrio parahaemolyticus* from aquatic food based on target-cyclized RCA with dynamic adapter followed by LAMP. *Foods*, *11*, 352. https://doi.org/10.3390/foods11030352

Zhang, M., Liu, C., Shi, Y., Wu, J., Wu, J., & Chen, H. (2020). Selective endpoint visualized detection of *Vibrio parahaemolyticus* with CRISPR/Cas12a assisted PCR using thermal cycler for on-site application. *Talanta*, *214*, 120818. https://doi.org/10.1016/j.talanta.2020.120818

Zhang, M., Liu, J., Shen, Z., Liu, Y., Song, Y., Liang, Y., Li, Z., Nie, L., Fang, Y., & Zhao, Y. (2021). A newly developed paper embedded microchip based on LAMP for rapid multiple detections of foodborne pathogens. *BMC Microbiology*, *21*, 197. https://doi.org/10.1186/s12866-021-02223-0

Zhang, Y., Yang, Q., Li, C., Yuan, Y., & Zhang, W. (2019). Sensitive and visual detection of *Cronobacter* spp. in powdered infant formula by saltatory rolling circle amplification method. *LWT*, *107*, 41–48. https://doi.org/10.1016/j.lwt.2019.02.050

Zhi, S., Shen, J., Li, X., Jiang, Y., Xue, J., Fang, T., Xu, J., Wang, X., Cao, Y., Yang, D., Yao, Z., & Yu, D. (2022). Development of recombinase-aided amplification (RAA)-exo-probe and RAA-CRISPR/Cas12a assays for rapid detection of *Campylobacter jejuni* in food samples. *Journal of Agricultural and Food Chemistry*, *70*, 9557–9566. https://doi.org/10.1021/acs.jafc.2c02581
